# Multimodal quantitative MRI finds early brain changes in asymptomatic X-linked adrenoleukodystrophy

**DOI:** 10.1093/braincomms/fcag311

**Published:** 2026-08-29

**Authors:** Kolja Meier, Prativa Sahoo, Lara Maleen Marten, Irini Gkalimani, Gunther Helms, Peter Dechent, Hendrik Rosewich, Steffi Dreha-Kulaczewski, Jutta Gärtner

**Affiliations:** Department of Pediatrics and Adolescent Medicine, University Medical Center Goettingen, Goettingen 37075, Germany; German Center for Child and Adolescent Health (DZKJ), Partner Site Goettingen, Goettingen 37075, Germany; Department of Pediatrics and Adolescent Medicine, University Medical Center Goettingen, Goettingen 37075, Germany; Department of Pediatrics and Adolescent Medicine, University Medical Center Goettingen, Goettingen 37075, Germany; Department of Pediatrics and Adolescent Medicine, University Medical Center Goettingen, Goettingen 37075, Germany; Department of Clinical Sciences Lund, Lund University, Lund 221 85, Sweden; German Center for Child and Adolescent Health (DZKJ), Partner Site Goettingen, Goettingen 37075, Germany; Department of Cognitive Neurology, MR Research in Neurosciences, University Medical Center Goettingen, Goettingen 37075, Germany; Department of Pediatrics and Adolescent Medicine, University Medical Center Goettingen, Goettingen 37075, Germany; German Center for Child and Adolescent Health (DZKJ), Partner Site Goettingen, Goettingen 37075, Germany; Department of Neuropaediatrics, University Hospital Tuebingen, Tuebingen 72076, Germany; Department of Pediatrics and Adolescent Medicine, University Medical Center Goettingen, Goettingen 37075, Germany; German Center for Child and Adolescent Health (DZKJ), Partner Site Goettingen, Goettingen 37075, Germany; Department of Pediatrics and Adolescent Medicine, University Medical Center Goettingen, Goettingen 37075, Germany; German Center for Child and Adolescent Health (DZKJ), Partner Site Goettingen, Goettingen 37075, Germany

**Keywords:** adrenoleukodystrophy, multimodal imaging, quantitative MRI, biomarker, neurodegeneration

## Abstract

Adrenoleukodystrophy (ALD) is an X-linked recessive disease caused by defects in the *ABCD1* gene, leading to the accumulation of very long-chain fatty acids in the nervous system, adrenal glands, blood and other tissues. Male ALD patients may present with either the severe neurological phenotype of cerebral ALD, characterized by inflammatory demyelination in the white matter and rapid neurological deterioration, or milder forms, such as adrenomyeloneuropathy. We aimed to identify early brain changes in asymptomatic ALD patients prior to the clinical onset of cerebral ALD or adrenomyeloneuropathy by exploratory analysis of multimodal MRI data. Multimodal MRI data—including structural images, diffusion tensor imaging, magnetization transfer imaging and MR spectroscopy—were obtained from asymptomatic ALD patients during regular follow-up MRIs. Longitudinal MRI data were analysed from 12 asymptomatic ALD patients aged 10–18 years who did not develop a cerebral ALD phenotype during our surveillance and from 12 healthy age-matched male controls. Volumetric analyses of brain structures were performed using structural MRI data, as well as region-of-interest measurements on diffusion tensor imaging, magnetization transfer imaging and MR spectroscopy data. Statistical analysis was performed using linear mixed-effects models. Volumetry indicated increased white matter volume in asymptomatic ALD patients. Diffusion tensor imaging analysis revealed increased values of radial and mean diffusivity in the supratentorial white matter. Magnetization transfer saturation values derived from magnetization transfer imaging metrics were increased in the cortical and subcortical grey matter, but not in the white matter. MR spectroscopy showed increased inositol levels in the frontal white matter. Our diffusion tensor imaging and MR spectroscopy data indicate early white matter pathology in adolescent asymptomatic ALD patients. Among these findings, increased white matter volume and, in addition, elevated magnetization transfer saturation values in the grey matter are novel MRI phenotypes associated with asymptomatic ALD. These results suggest that not only white matter but also grey matter can be pathologically altered even before the onset of neurological phenotypes. Multimodal MRI parameters therefore constitute promising biomarkers for the assessment of pre-symptomatic brain tissue alterations in ALD and may be useful in future clinical studies targeting early therapeutic intervention.

## Teaser

Meier et al. applied multimodal MRI in asymptomatic adolescent adrenoleukodystrophy patients and identified early brain alterations. Increased white matter volume, elevated diffusivity and inositol, and abnormal magnetization transfer indicate pre-symptomatic white and grey matter involvement. These findings highlight multimodal MRI as a promising tool for monitoring disease progression in adrenoleukodystrophy.

## Graphical Abstract

**Graphical Abstract:**
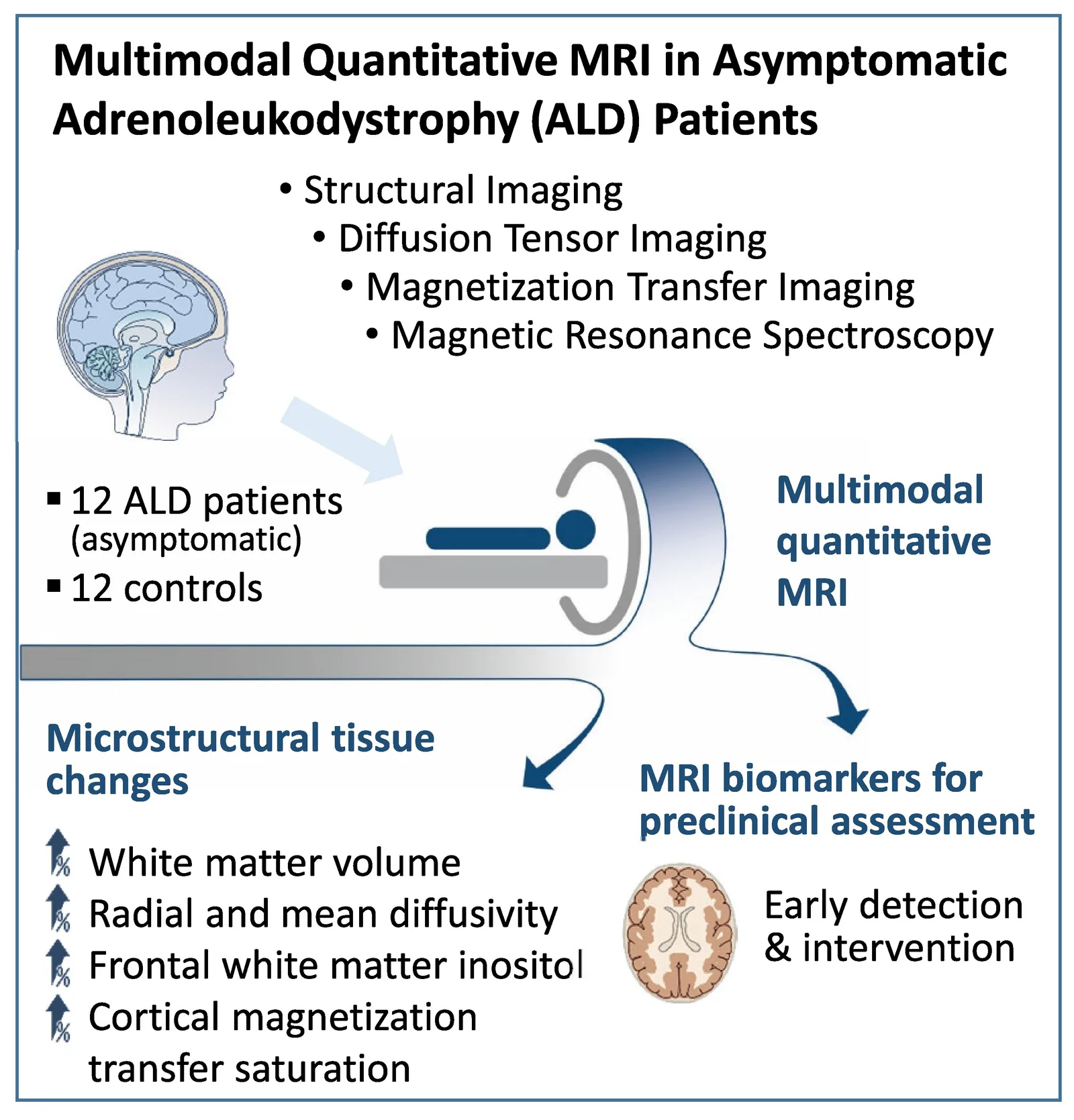

## Introduction

X-linked adrenoleukodystrophy (ALD) is an X-linked recessive disease caused by defects in the *ABCD1* gene, leading to the accumulation of very long-chain fatty acids (VLCFAs) in the nervous system, adrenal glands, blood and other tissues. Almost all hemizygous males with *ABCD1* pathogenic variants develop adrenomyeloneuropathy (AMN), a disorder marked by adrenocortical insufficiency, progressive spinal cord and peripheral nerve dysfunction later in life.^1^ Cerebral adrenoleukodystrophy (CALD) is caused by inflammatory demyelination in the cerebral white matter (WM) of the brain, leading to rapid neurological deterioration. CALD has its peak in male children between the ages of 3 and 12 years, but can also occur in AMN patients.^2,3^ The molecular pathology that underlies demyelination in CALD or neurodegeneration in AMN is still not well understood. Fundamental questions, such as whether myelin or axon structures are the first to be affected in the disease course, remain controversial.^4,5^ Mouse models have been studied extensively in the past. However, they do not accurately reflect the human phenotype, as they do not undergo spontaneous demyelination but merely show an increased susceptibility to experimental autoimmune encephalomyelitis or cuprizone-induced demyelination.^6–9^ Furthermore, for neuropathological studies, brain or spinal cord tissue from autopsies is usually only available from patients with end-stage disease and is therefore unsuitable to identify early pathological changes in brain tissue. Therefore, MRI is the key method for analysing cerebral tissue alterations in the early stages of ALD.

Supratentorial microstructural tissue changes in AMN patients who do not show clinical signs of cerebral disease and have normal MRI results were identified and characterized through multiparametric quantitative MRI studies. Diffusion tensor imaging (DTI) revealed increased radial diffusivity (RD) and decreased fractional anisotropy (FA), indicating early microstructural abnormalities of the myelin sheath.^5,10–12^ MR spectroscopy (MRS) showed a reduced total N-acetyl aspartate/creatine ratio (tNAA/tCr), reflecting axonal damage in the cerebral WM. Furthermore, elevated inositol-to-creatine (Ins/tCr) ratios and choline-to-creatine (Cho/tCr) ratios were described. Ins and Cho are commonly considered markers for gliosis and increased membrane turnover, respectively, and increase in conditions, such as active demyelination, inflammation or cell proliferation.^13–15^ These findings raise the question of whether the pathological changes found in AMN are already present at a clinically asymptomatic stage, allowing early detection of a potential predisposition to CALD.

We diagnose and closely monitor our ALD patients using standard molecular genetic tests, metabolic assessments and MRI. Our MRI protocol includes the parameters recently recommended for the screening and routine monitoring of ALD patients.^16^ All patients included in this study were asymptomatic; they did not have inflammatory brain lesions suggestive of CALD, and they also showed no or only subtle clinical signs of AMN. Multimodal MRI studies of the brain were performed regularly to track disease course and to monitor for signs of CALD. The imaging protocol comprised structural MRI sequences, DTI, magnetization transfer imaging (MTI) and MRS. To characterize early pathological changes in the brain tissue of asymptomatic ALD (AALD) patients, we performed volumetry on structural MRIs, took atlas-based region-of-interest measurements on DTI and MTI data, and analysed MRS spectra from the frontal and occipital WM.

## Materials and methods

### Patient cohort

Male patients diagnosed with ALD either through molecular genetic or metabolic analyses, or both (e.g. through family testing or following an adrenal crisis), were included in this study. All subjects were monitored for the development of CALD by brain MRI at 6-month intervals in the Division of Pediatric Neurology, Department of Pediatrics and Adolescent Medicine, University Medical Center Goettingen, Germany. The data were collected from 2005 to 2018. MRI scans were conducted under sedation if necessary. Only MRI data from patients aged 10–18 years who did not develop CALD during the entire surveillance at our centre were included.

The patients’ MRI data were compared with age-matched male healthy control subjects. For site-specific parametrization to the braincharts.io centiles, we included additional data from healthy female adolescent controls. All MRI investigations of the controls were performed without sedation.

The local institutional Ethics Committee approved the study (19/12/03, 6/6/05, 2/11/12), and informed written consent was obtained from all families according to the Declaration of Helsinki.

### MRI acquisition

All MRI scans were performed as previously described using the same 3 T clinical MR scanner (Siemens Healthineers, Erlangen, Germany).^17^ Necessary upgrades were implemented during the follow-up period (Siemens Magnetom TRIO to Magnetom Tim TRIO). For volumetric, DTI and MTI analyses, only data acquired on the Magnetom Tim TRIO were used. For MRS analyses, data from both scanner versions were used. Appropriate routine quality assurance measures within the MR facility ensured the continuous comparability of the MRI data throughout the study. Not all imaging modalities were acquired consistently across MRI sessions, with only a subset of modalities available for the majority of sessions. Details about the MRI acquisition parameters for structural imaging, DTI, MTI imaging, MT saturation (MTsat) mapping and MRS are described in the Supplementary Methods.

### Data analysis

#### MRI volumetry

For volumetric analyses, only images recorded on the Magnetom Tim TRIO system were included. We used the FreeSurfer toolbox version 6 for brain segmentation. Quality control included visual assessment based on established rating criteria^18^ and exclusion of scans with more than 100 surface holes in the FreeSurfer parcellation. Further details about the volumetric analysis and its alignment with an open-source reference dataset are described in the Supplementary Methods.

#### Centiles with brainchart.io

To calculate volume centiles for WM, cortical grey matter (CGM) and subcortical grey matter (SGM) and ventricles, we utilized the website https://brainchart.shinyapps.io/brainchart/.^19^ A total of 34 T1-weighted images from 16 healthy female participants (17 recordings, 1–2 recordings per subject) and 13 healthy male participants (17 recordings, 1–3 recordings per subject) were used for site-specific parameterization.

#### Diffusion MRI analysis

For diffusion MRI analysis, we used only images obtained on the Magnetom Tim TRIO system. Pre-processing of diffusion images was carried out using the standard pipeline of QSIPrep version 0.20.1 without b1 bias correction.^20^ A detailed description of the pre-processing steps using the QSIprep toolbox is given in the Supplementary Methods. The reorientation of the pre-processed images to FSL standard space and the creation of a brain mask were performed using QSIRecon (reorient_fslstd, part of the QSIprep 0.20.1 toolbox). DTIfit from the FSL toolbox (version 6.0.7) was employed to independently fit a diffusion tensor to each voxel, yielding voxel-wise maps of the DTI metrics FA, mean diffusivity (MD), axial diffusivity (AD) and RD for each subject. Quality control of the DTI data was conducted through visual inspection and QSIprep quality metrics. The tract-based spatial statistics workflow contained in FSL (version 6.0.7) was adapted to register the JHU ICBM-DTI-81 atlas to the DTI images.^21–23^ Average DTI measures for supratentorial WM tracts and major tract classes were obtained with fslmaths. Further details are described in the Supplementary Methods.

#### Magnetization transfer imaging

For MTI analysis, we used only images obtained on the Magnetom Tim TRIO system. Parameter maps of MTsat were generated as previously described and registered to the FreeSurfer segmentations.^17^ The overlay of MTsat maps with the segmentations was visually assessed. Region-wise median MTsat values were obtained using fslstats. Further details on the MTsat analysis are described in Supplementary Methods.

#### Spectroscopy analysis

From 1H MRS data, absolute concentrations (in mmol/L) of N-acetylaspartate and N-acetylaspartylglutamate (tNAA), creatine and phosphocreatine (tCr), choline-containing compounds (Cho) and myo-inositol (Ins) were estimated by using LCModel.^24^ Metabolite concentrations from the frontal WM and parieto-occipital (PO) WM were included in the statistical analysis. Average values for metabolites were calculated when MRS was measured in the left and right hemispheres. For quality control, we excluded recordings with outlier values in two or more of the four analysed metabolites. A metabolite measurement is considered an outlier if it falls below the first quartile by 1.5 interquartile range (IQR) or above the third quartile by the same amount. Outlier data points of single metabolites were excluded from further analysis of the respective metabolite.

### Statistical analysis

Statistical comparisons of the volumetry data were performed using R version 4.2.1 (https://www.R-project.org/). We employed the ‘lmerTest’ package for linear mixed-effects model analysis, the ‘performance’ package for model performance analysis and the ‘rstatix’ package for the Wilcoxon signed-rank test. Further details about the specific tests and model parameters can be found in the Supplementary Methods. False discovery rate (FDR) corrected q-values were obtained with the Benjamini–Hochberg procedure.

## Results

### Description of the patient cohort

We analysed multimodal MRI data from 12 patients with ALD aged between 10 and 18 years. Of the 12 patients, nine were followed up to the age of 17 years or older. Supplementary Table 1 refers to clinical details. None of the patients showed clinical or radiographic signs of CALD during the surveillance period. With the exception of one patient, all patients suffered from adrenocortical insufficiency and required corticosteroid and mineralocorticoid replacement therapy. Two patients displayed minor pyramidal tract signs, and one of them was suspected of developing a voiding disorder. These could be considered signs of early AMN. Somatosensory potentials were abnormal in both patients. However, both had a normal gait and reported no sensory disturbances. Since AMN typically begins between the ages 20 and 30 and no major clinical indicators of progressive spinal cord disease were observed, both patients were included in the analysis.

### Volumetry analysis

#### Major brain segments

We conducted quality control on structural T1-weighted images of 12 control subjects (16 recordings, 1–4 recordings per subject) and 12 AALD patients (101 recordings, 1–15 recordings per subject). After quality control, volumetric data of 10 male control subjects (12 recordings, 1–2 recordings per subject) and 12 AALD patients (88 recordings, 1–14 recordings per subject) were used for further analysis.

**Figure 1:**
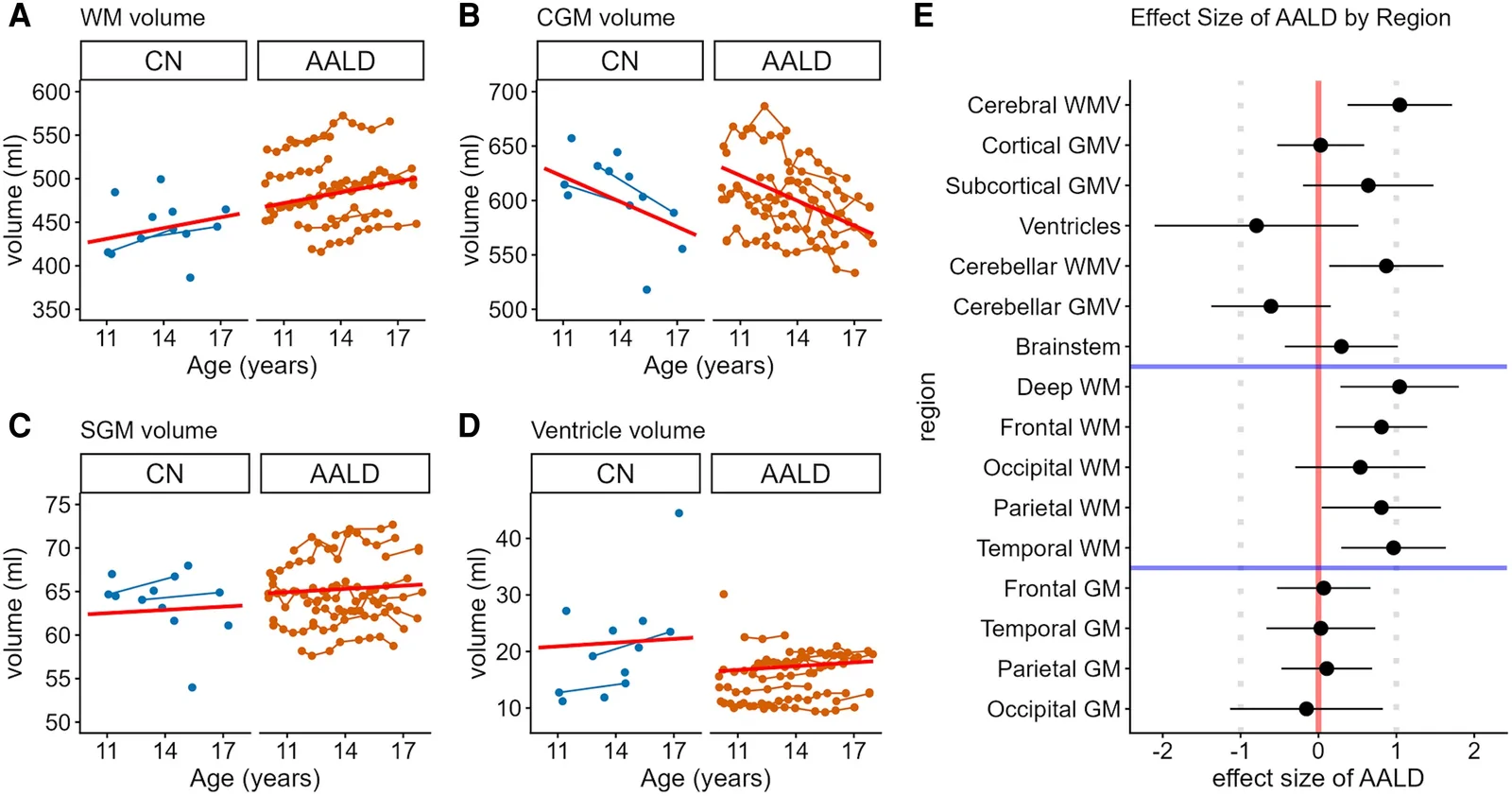
**Volumetry analysis. (A–D)** Longitudinal volume measurements of WM (**A**), CGM (**B**), SGM (**C**) and ventricles (**D**) in controls (CN, *n* = 10 subjects, 12 recordings, 1–2 recordings per subject) and AALD patients (*n* = 12 subjects, 88 recordings, 1–14 recordings per subject), shown in millilitre. Each dot represents a measurement. Connected dots indicate repeated measurements from the same individual. The solid line illustrates the group-specific intercept and the age slope of the linear mixed-effects model analysis. **(E)** Effect sizes of AALD versus control group for brain structure volume by linear mixed-effects model analysis. Data were z-normalized for linear mixed-effects model analysis. Error bars indicate 95% CI of the effect size estimate. The vertical line at zero indicates no change; positive/negative values indicate increased/decreased volumes in AALD relative to controls. The results of the linear mixed-effects model analysis are shown in Supplementary Table 2. GMV = grey matter volume, WMV = white matter volume.

Cerebral WM volume (WMV; WM refers to cerebral WM) increased with age during adolescence across all subjects, while cortical GM volume (GMV) physiologically declined. Subcortical GMV (sGMV), ventricle volume, cerebellar GMV and brainstem volume also increased with respect to age. Estimated total intracranial volume (eTIV) was a significant predictor of WMV, GMV, sGMV, cerebellar GMV and WMV as well as brainstem volume. Linear mixed-effects modelling revealed a significant increase in cerebral WMV in AALD patients compared to healthy controls [effect size estimate 1.04, 95% confidence interval (CI) 0.37–1.73, *t* = 3.27, *P* = 0.004, *q* = 0.03]. Model fit was good, with a marginal *R*^2^ of 0.28 and a conditional *R*^2^ of 0.98. Cerebellar WMV showed a trend towards increase in AALD patients; however, this effect did not remain significant after FDR correction (effect size estimate 0.87, 95% CI 0.14–1.60, *t* = 2.46, *P* = 0.02, *q* = 0.07). No significant group differences were observed for cortical GMV, sGMV or ventricular volume (Fig. 1; Supplementary Table 2 and Fig. 1).

We further compared our volumetric measurements with an external dataset.^25^ To analyse for batch effects, we first analysed only healthy control data with a linear model. We found significant batch effects in healthy controls for eTIV and cortical GMV, but not for WMV, sGMV and ventricular volume. These findings suggest that batch effects predominantly affect cerebral surface and outer CSF spaces (Supplementary Table 3). Therefore, eTIV was not included as a covariate in the linear mixed-effects models comparing AALD patients with the external datasets, despite its known value as a predictor of WMV and its potential to improve model performance.

Nevertheless, WMV—but not GMV, sGMV or ventricular volume—remained significantly increased when comparing AALD patients with the external datasets using a linear mixed-effects model (effect size estimate 0.92, 95% CI 0.07–1.77, *t* = 2.14, *P* = 0.035; Supplementary Fig. 2 and Table 4).

#### Regional white matter and grey matter volumes

Deep, frontal and temporal WMV were significantly increased in AALD patients, while parietal WMV was only moderately increased at the threshold of statistical significance after multiple testing correction. Occipital WMV was not significantly altered in AALD patients (statistical test results in Supplementary Table 2). There were no regional differences in cortical GMV. The ventral diencephalon and hippocampus were moderately increased in AALD patients; however, both findings did not remain significant after multiple testing correction (Fig. 1; Supplementary Figs. 3–6 and Table 2).

#### Volume centile analysis

**Figure 2:**
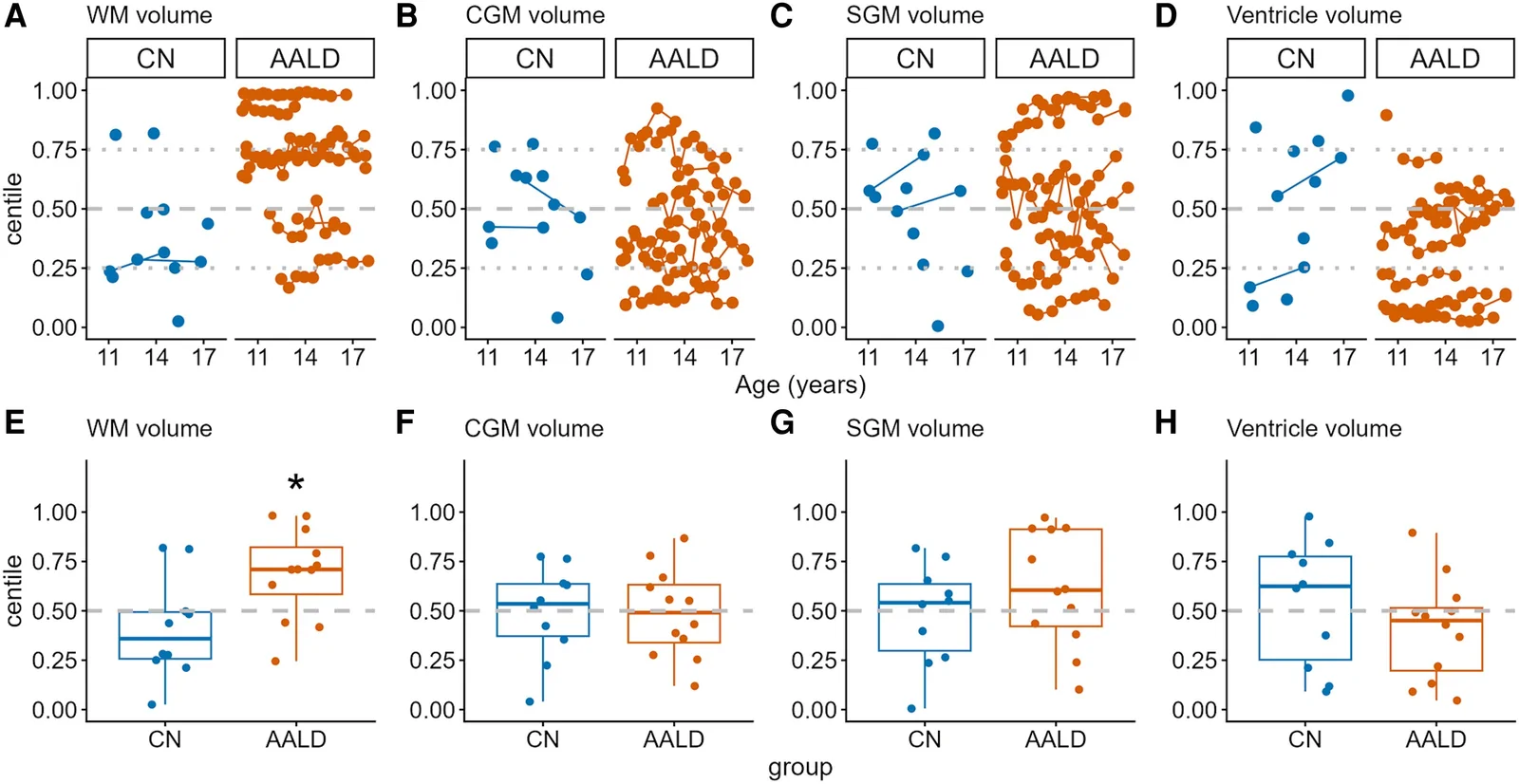
**Volume centile analysis. (A–D)** Longitudinal centiles for WM (**A**), CGM (**B**), SGM (**C**) and ventricle volumes (**D**) in controls (CN, *n* = 10) and AALD patients (*n* = 12) calculated by *braincharts.io.* Each dot represents an individual measurement. Connected dots indicate repeated measurements from the same individual. No statistical test was performed on **A–D**. (**E–H)** Boxplots showing the median centiles for WM (**E**), CGM (**F**), SGM (**G**) and ventricles (**H**). Each dot represents one subject, calculated as the median of all their centile measurements. Asterisk indicates the *P*-value (* = *P* < 0.05) from a one-sample Wilcoxon signed-rank test against the group median of 0.5. Boxplots show the median (line within the box), the IQR (box limits = 25th and 75th percentiles) and whiskers extending to the smallest and largest values within 1.5 × IQR from the lower and upper quartiles. Points beyond the whiskers represent outliers. The Wilcoxon signed-rank test showed a significant difference from 0.5 for WM centiles in AALD patients (W = 67, *P* = 0.027) but not for controls (W = 17, *P* = 0.32). CGM centiles (CN: W = 27, *P* = 1; AALD: W = 36, *P* = 0.85), SGM centiles (CN: W = 28, *P* = 1; AALD: W = 57, *P* = 0.18) and ventricle centiles (CN: W = 30, *P* = 0.84; AALD: W = 21, *P* = 0.18) were not significantly altered in both groups.

The WMV in AALD patients consistently remained on one centile, suggesting that there is no disproportionate growth of WM during adolescence. The average WMV centile for the AALD group was significantly above 0.5, the expected median of a standard population sample (one-sample Wilcoxon signed-rank test against a median of 0.5, AALD: median = 0.709, 95% CI 0.54–0.85, *P* = 0.027; control: median = 0.382, 95% CI 0.24–0.62, *P* = 0.32). The WMV centiles of the control group, as well as the volume centiles for cortical GM, subcortical GM and ventricles of both groups, did not significantly differ from 0.5 when tested in a similar way (Fig. 2).

### Diffusion tensor imaging analysis

Quality control was performed on DTI data from 9 controls (11 recordings, 1 subject with 3 recordings) and 7 AALD patients (22 recordings, 1–5 recordings per patient). After quality control, data from 5 controls (7 recordings, 1 subject with 3 recordings) and 6 AALD patients (20 recordings, 1–5 recordings per patient) were analysed. The DTI recordings did not sufficiently cover the infratentorial WM across all subjects; therefore, these infratentorial WM tracts were excluded from the analysis.

We used a standard FA atlas for segmenting the DTI data and extracted tract-wise the diffusion parameters: FA, AD, RD and MD. In supratentorial WM, linear mixed-effects modelling showed that RD and MD were significantly increased in AALD patients compared with healthy controls (RD: effect size estimate 1.27, 95% CI 0.27–2.28 *t*-value 3.17, *P*-value 0.022; MD: effect size estimate 1.22, 95% CI 0.71–1.72, *t* = 4.96, *P* = 0.00004). The model for supratentorial RD indicated a good fit (marginal *R*^2^ 0.55, conditional *R*^2^ 0.88). The linear mixed-effect model for MD failed to converge, as the estimated variance for the random intercept of subjects was 0. Nevertheless, the marginal *R*^2^ of 0.69 indicated a good model fit for fixed effects. This indicated that, after accounting for fixed effects, there was no detectable between-subject variability in MD. A corresponding linear model analysis for supratentorial MD showed similar estimates for the fixed effects, supporting the robustness of the finding.

**Figure 3:**
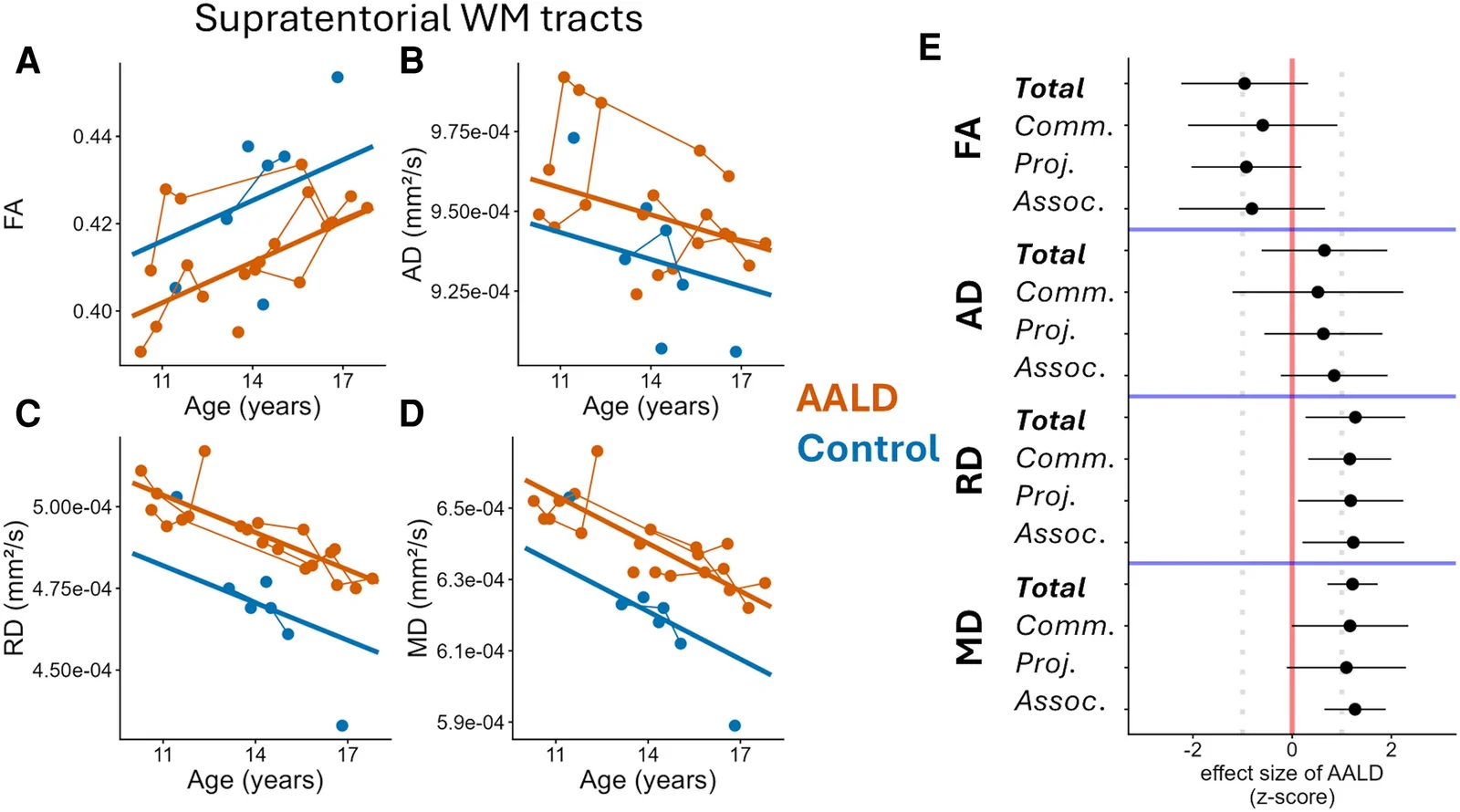
**Diffusion imaging analysis. (A–D)** Longitudinal measurement values from control subjects (*n* = 5, 7 recordings, 1 subject with 3 recordings) and AALD patients (*n* = 6, 20 recordings, 1–5 recordings per patient) of diffusion MRI parameters FA (**A**), AD (**B**), RD (**C**) and MD (**D**) in the supratentorial WM. Each dot represents a measurement. Connected dots with thin lines indicate repeated measurements from the same individual. The thick lines illustrate the group-specific intercepts and the age slope of the linear mixed-effects model analysis. **(E)** Effect size of AALD versus controls for diffusion parameters (FA, MD, RD, AD), measured across all supratentorial WM tracts (Total) and separately for commissural (Comm.), projection (Proj.) and association (Assoc.) tracts, estimated by linear mixed-effects model analysis. The data were z-normalized for linear mixed-effects model analysis. Error bars indicate 95% CI of the effect size estimate. The vertical line at zero indicates no change, and positive/negative values indicate an increase/decrease in AALD patients compared to controls. Full model effect estimates are listed in Supplementary Tables 5 and 6.

We then performed a linear mixed-effect model analysis of DTI measurements in major WM tract classes (commissural, projection, association). RD was significantly increased in AALD patients in all three tract classes. MD was significantly altered in association tracts and showed a strong trend towards significance in commissural and projection tracts. No clear preferential involvement of any tract class was observed. No significant alterations were detected in FA or AD (Fig. 3; Supplementary Tables 5 and 6 and Fig. 7).

### Magnetization transfer imaging

**Figure 4:**
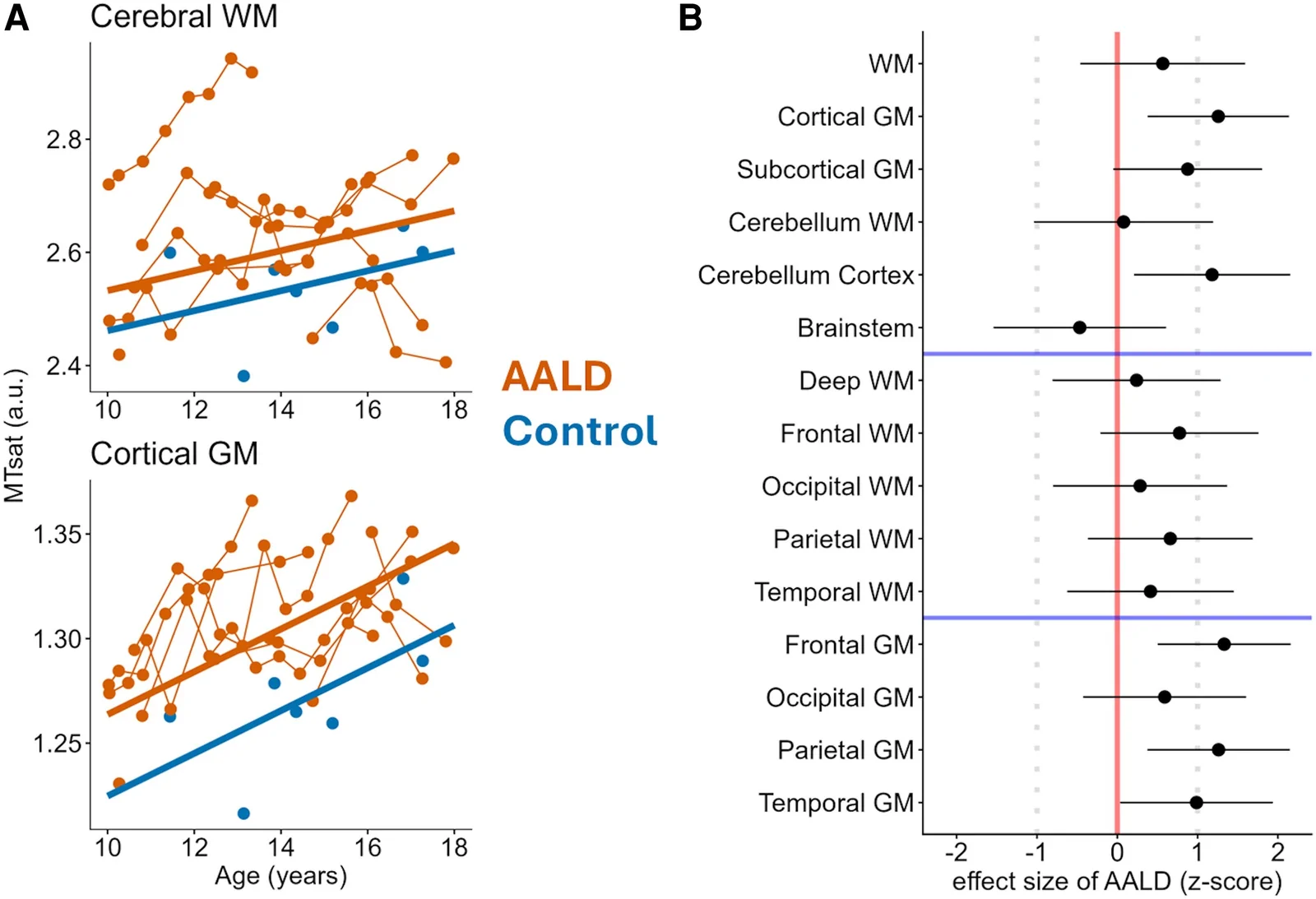
**MTI analysis. (A)** Longitudinal MTsat values in the cerebral WM and CGM for AALD patients (*n* = 10, 53 recordings, 1–11 recordings per subject) and controls (*n* = 7, 7 recordings) from age 10 to 18 years. Each dot represents a measurement. Connected dots indicate repeated measurements from the same individual. The thick lines illustrate the group-specific intercepts and the age slope of the linear mixed-effects model analysis. (**B)** Effect sizes of AALD versus control group on MTsat, estimated by linear mixed-effects model analysis. The data were z-normalized for linear mixed-effects model analysis. Error bars indicate the 95% CI of the effect size estimate. The vertical line at zero indicates no change, and positive/negative values indicate an increase/decrease in AALD patients as compared to controls. Full model effect estimates are listed in Supplementary Table 7.

After appropriate quality control, we used MTsat maps from 7 control subjects (7 recordings) and 10 AALD patients (53 recordings, 1–11 recordings per subject) for further analysis. There were no significant differences in global and regional WM MTsat values between the control group and AALD. However, MTsat was significantly increased in the cerebral cortical GM in AALD patients compared to control subjects (effect size estimate 1.26, 95% CI 0.38–2.14 *t* = 3.0, *P* = 0.008, *q* = 0.045). The *R*^2^ values for cortical GM indicated a sufficient model fit (marginal *R*^2^ 0.43, conditional *R*^2^ 0.74). In the regional cortical analysis, MTsat was significantly increased in the frontal and parietal cortex, while the temporal cortex showed a moderate increase that was not significant after multiple testing correction, and no significant increase was observed in the occipital cortex. We also found moderate regional subcortical MTsat increases in the caudate nucleus, putamen and thalamus, as well as a moderate MTsat increase in the cerebellar cortex; however, none of those remained significant after multiple testing correction (Fig. 4; Supplementary Table 7 and Figs. 8 and 9).

### MR spectroscopy

WM MRS data of the frontal lobe (FL) and PO areas of control subjects (PO: 12 subjects, FL: 7 subjects, each 1 recording per subject) and AALD patients (PO: 11 subjects, 1–13 recordings per subject, 93 recordings total; FL: 10 subjects, 2–12 recordings per subject, 80 recordings total) were processed. In the FL, MRS data of all control subjects and two AALD patients included in the analysis were recorded before the upgrade of the MR scanner, while most recordings of AALD patients were performed after the scanner upgrade. For PO, the MRS data of three control subjects were recorded after the update of the scanner, and only two AALD patients were recorded before the scanner upgrade. This imbalance could be a potential source of bias, although previous reports could show that MRS measures are not significantly affected by MR upgrades.^26^ To directly assess potential scanner upgrade effects, we compared the last pre-upgrade and first post-upgrade scans in AALD patients using paired *t*-tests, including additional recordings outside the age range of this study. We found that no metabolite was significantly affected by the scanner upgrade (Supplementary Fig. 10 and Table 8). Therefore, we did not include the MRI upgrade as a covariate in the linear mixed-effects models. During quality control, we excluded one MRS recording from an AALD patient due to outliers in more than one metabolite. We excluded three Cho, six Ins, two tCr and four tNAA outlier values measured in the PO WM and three Cho, one Ins, four tCr and one tNAA outlier values measured in the frontal WM from further analysis.

**Figure 5:**
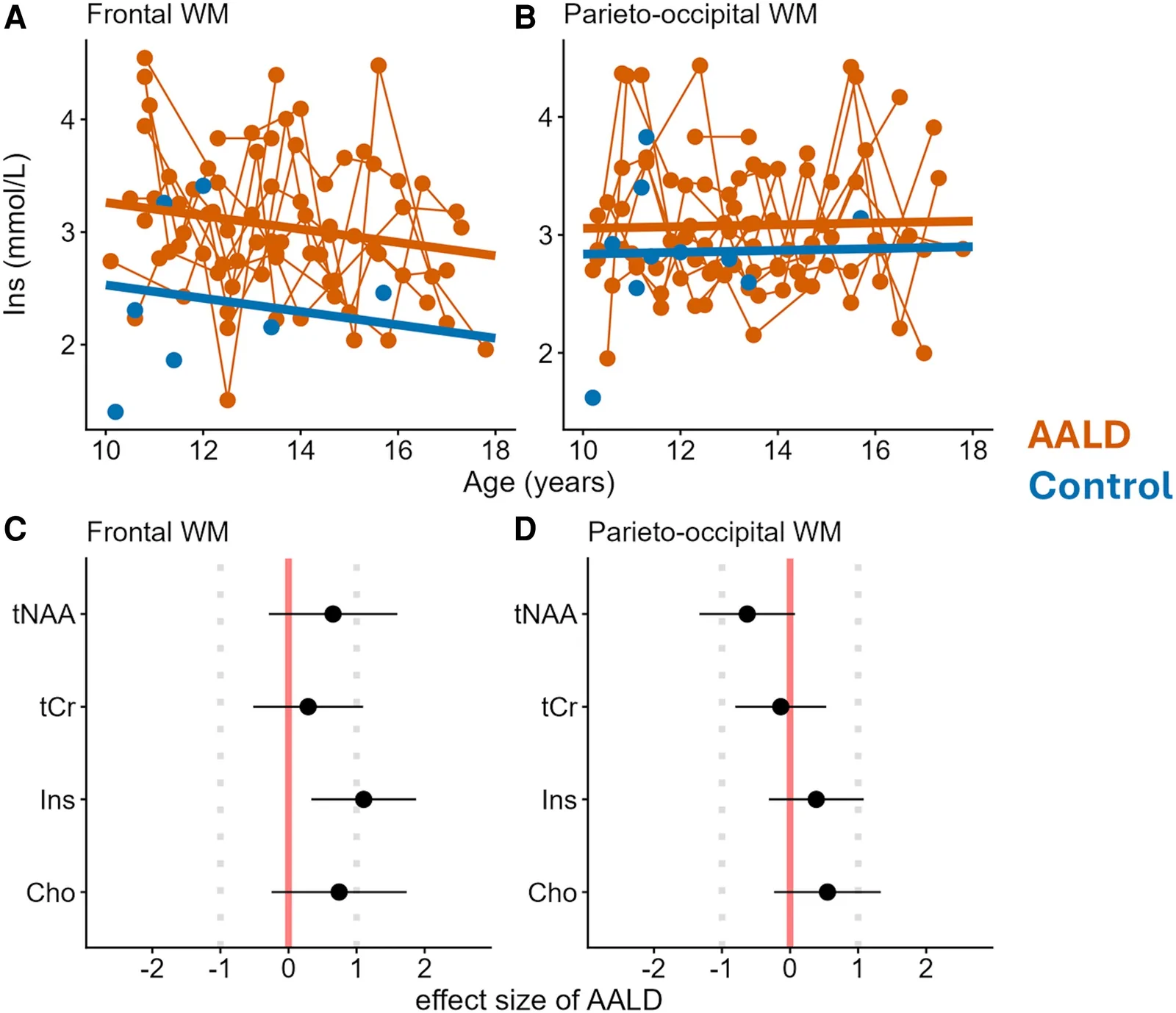
**MRS analysis.** (**A and B**) Longitudinal Ins values for AALD patients and controls measured in the cerebral frontal (**A**, 7 controls, 10 AALD patients) and PO (**B**, 10 controls, 11 AALD patients) WM from age 10 to 18 years. Each dot represents a measurement. Connected dots indicate repeated measurements from the same individual. The thick lines illustrate the group-specific intercepts and the age slope of the linear mixed-effects model analysis. (**C and D**) Effect sizes of AALD versus control group for total N-acetyl aspartate (tNAA), total creatine (tCr), choline (Cho) and Ins in the frontal (**C**) and PO (**D**) WM using a linear mixed-effects model analysis. The data were z-normalized for linear mixed-effects model analysis. Error bars indicate 95% CI of the effect size estimate. The vertical line at zero indicates no change, and positive/negative values indicate an increase/decrease of metabolite levels in AALD compared to control. Full model effect estimates are listed in Supplementary Table 9.

The linear mixed-effects model analysis showed a significant increase in Ins in the frontal WM of AALD patients (effect size estimate 1.1, 95% CI 0.33–1.87, *t* = 2.85, *P* = 0.005, *q* = 0.022), while the other parameters were not significantly altered. The linear mixed-effects models for Ins and tCr in the frontal WM did not converge, and the estimated variance for the random intercept of subjects was zero for both. The marginal *R*^2^ values (0.02–0.10) indicated a weak model fit for fixed effects across all MRS analyses (Fig. 5; Supplementary Table 9 and Fig. 11).

## Discussion

At our centre, ALD patients are monitored clinically and neuroradiologically every 6–12 months from the day of their diagnosis, in accordance with international consensus guidelines.^1^ The multimodal MR protocol used facilitates the analysis of extensive quantitative MRI-derived measurements. In this study, we focused on clinically healthy adolescent AALD patients aged 10–18 years to identify alterations that could indicate early microstructural or metabolic disturbances in brain tissue. None of them showed abnormalities in structural cranial MRI, nor did they develop the CALD phenotype during the follow-up period. Abnormalities were identified in these AALD patients using various MR modalities, indicating early changes in axonal and myelin structures and glial cells within the WM and also the GM.

### Histopathological characteristics in cerebral adrenoleukodystrophy

We first compared our *in vivo* results with known histopathological studies of CALD. Histopathology is well described in CALD, but is based on post-mortem tissue samples from patients, often in the end stages of disease. Histopathological analysis of normal-appearing white matter (NAWM) in CALD revealed an increased number of microglia expressing homeostatic markers, while myelin, axons and astrocytes appeared normal.^27–29^ Leukodystrophy lesions are characteristically discriminated into four zones, three of which are detectable with conventional MRI. In the prelesional zone, which appears as T2 hyperintensity without contrast enhancement, normal axonal morphology was observed with merely immunohistological signs of damage, accompanied by mild signs of myelin affection, occasional reactive astrocytes and almost complete absence of microglia. In the contrast-enhancing, actively demyelinating zone, there is a massive inflammatory reaction, parallel to myelin breakdown, extensive axonal damage, microgliosis and reactive astrocytes with the beginning of gliosis. In the gliotic core region, myelin and axons are almost entirely absent.

These results demonstrate that in CALD, inflammation and demyelination are associated with more widespread alterations in brain tissue, also affecting areas beyond the lesions. Whether the histopathological findings in NAWM in CALD are comparable to those in adolescent AALD patients cannot yet be conclusively answered.

### Diffusion tensor imaging and myelin-sensitive magnetization transfer imaging suggest dysmyelination in asymptomatic adrenoleukodystrophy

Analyses of DTI parameters indicated a significant increase in RD and MD in supratentorial WM. Both the linear mixed-effects model applied for RD and the linear model used for MD showed a good model fit. Decreased FA together with increased RD or MD has previously been described in the cerebral WM of AMN patients,^5,10–12^ as well as in the NAWM of CALD patients.^30–32^

Each of the DTI parameters corresponds to different WM properties. The most frequently used DTI parameter, FA, is considered a rather non-specific indicator of the microstructural integrity of both axons and myelin and typically decreases upon demyelination, inflammation and axonal loss. RD measures diffusion perpendicular to axons and indicates the myelination status and integrity of the myelin sheaths. Increased RD values are particularly linked to demyelination. In contrast, AD reflects diffusion parallel to axons, but the clinical implications of changes in AD are less well understood.^33,34^

Myelin abnormalities have been discussed as an underlying pathological mechanism for the diffusion abnormalities seen in AMN.^10^ In the case of demyelination, a decrease in the myelin-sensitive measure MTsat is to be expected. A quantitative MRI study described a decrease in the myelin-sensitive measure magnetization transfer ratio (MTR; comparable to the MTsat measure here) in CALD lesions, along with a decrease in FA and an increase in MD.^35^ In contrast, our results did not show significant MTsat alterations in the WM, suggesting that demyelination is unlikely in our cohort. Research involving mouse models of dysmyelination has demonstrated elevated RD in their WM, indicating that other myelin pathologies beyond pure loss may contribute both quantitatively and qualitatively to increased RD.^34,36^ Taken together, the DTI findings in conjunction with the myelin-sensitive MTsat in our AALD patient cohort are most consistent with dysmyelination and impaired myelin integrity. Notably, in agreement with our imaging data, there are a few neuropathological reports of adult AMN brains describing microscopic foci of dysmyelination relative to axonal and oligodendrocytic sparing.^37^

### Increased frontal MR spectroscopy inositol suggests glial cell activation in asymptomatic adrenoleukodystrophy

To analyse cerebral metabolites *in vivo* using MRS, voxels were positioned in the frontal and PO brain WM, targeting regions commonly affected by demyelinating lesions. The linear mixed-effects models used to analyse MRS data showed overall poor fit. Additionally, MRS recordings were performed across two MR scanner upgrades. Although our analyses indicated that metabolite measures were not affected by the scanner upgrade, and previous studies indicate that scanner upgrades do not substantially influence MRS measurements,^26^ this remains a potential source of variability that may affect the reliability of the MRS findings.

In the frontal WM, we observed a significant increase in Ins concentration in our AALD patient cohort compared to controls. This metabolite is considered an astrocyte marker, reflecting glial cell function, and may also serve as an important osmolyte.^38^ Elevated Ins levels have been consistently found in demyelinating lesions as well as in several inflammatory CNS diseases without apparent loss of myelin, suggesting concomitant microglial activation and gliosis.^39,40^ In line with our observations, increased Ins concentrations were reported in NAWM of CALD patients.^35^ Similar MRS patterns were also reported in the spinal cord of adult AMN patients and in the NAWM of AMN patients,^10,15^ suggesting that increased Ins is a characteristic feature of ALD.

While we can only speculate about the pathomechanisms underlying the increase in Ins, several studies on molecular markers support the notion that glial cells are activated in ALD. Importantly, microglia and astrocytes, which give rise to the Ins peak in MRS, are the cell types that predominantly express ABCD1 in the brain.^41^ In CALD, microglia are increased in the NAWM, particularly in areas adjacent to the prelesional zone^28,29^; however, it is unknown whether the characteristics of the NAWM in CALD are comparable to those in AALD. In cultured mouse astrocytes, silencing of the *ABCD1* and *ABCD2* genes leads to VLCFA accumulation and an inflammatory response.^42^ A study of astrocytes derived from human-induced pluripotent stem cells (hiPSC) from ALD patients revealed reactive transcriptional profiles and a dysregulated lipidome.^43^ A study on autopsy tissue from the brains of CALD patients indicated that astrocytic stress precedes myelin loss.^44^ Furthermore, glial fibrillary acidic protein—a marker for astrocyte activation—showed a trend to be increased in children and adolescents with AALD and was significantly increased in AMN patients.^45,46^ Taken together, our findings support the notion that glial cell activation occurs in the NAWM of AALD patients and is already present early in the course of the disease.

### Increased regional white matter volume and increased grey matter magnetization transfer saturation are new MRI phenotypes of asymptomatic adrenoleukodystrophy

The analysis of our quantitative imaging data revealed two previously undescribed MRI phenotypes for adolescent AALD. First, we found regionally increased WMV and, second, a regionally increased cortical MTsat compared to healthy controls. For both findings, the linear mixed-effects models showed a good fit to the data. Notably, both abnormal findings predominantly affect the FL, but not the occipital lobe.

Increased WMV determined by MR volumetry has been reported in children with autism spectrum disorder and developmental language disorder and adults with neurofibromatosis Type I or methamphetamine abuse.^47–49^ In all studies, the authors could only speculate about underlying mechanisms. Adaptive glial changes, altered WM integrity or differences in the degree of axonal packing in conjunction with altered myelination were discussed. Reduced axonal packing density has been shown to correlate with decreased FA and increased RD, consistent with our DTI results. Larger distances between axons could indeed contribute to an increase in WMV. Furthermore, the assumption of hypertrophic, reactive glial cells is supported by the elevated Ins concentrations in the MRS results. Overall, increased WMV appears to be a novel phenotype of AALD, to which multiple pathogenic processes likely contribute.

MTI is usually used for assessing WM and myelination. In contrast, we observed increased MTsat values in the patients’ cerebral cortex, while MTsat in the WM was not significantly altered. MTsat measures the macromolecular content of a tissue and is typically used to assess WM integrity; therefore, there are not many studies analysing MTI-derived measures in the cerebral cortex. Two studies focused on regional correlations of MTI parameters in the cortex with transcriptome data from the Allen Human Brain Atlas. One study found a correlation between the MTR and genes involved in dendritic growth.^50^ They concluded that MTR is also influenced by macromolecules associated with the cell membranes of the dendritic arbour. The other study found a correlation of MTsat with genes enriched in GABAergic neurons, glutamatergic neurons, endothelial cells and microglia.^51^ MTsat has been reported to be decreased in the normal-appearing cortical GM in multiple sclerosis, and MTR was decreased in Alzheimer’s disease patients.^52,53^ While MTsat reduction in multiple sclerosis patients was attributed to demyelination, the authors of the Alzheimer’s study linked the MTR reduction to the loss of dendritic spines. An increase in cortical MTsat, similar to that in our study, has been described in the left hemisphere of schizophrenia patients, and neuroinflammation and altered myelination have been discussed as underlying reasons.^54^

Up to now, we can only speculate about the underlying pathology of the cortical MTsat increase in AALD. An increased number of dendrites, resulting in a larger neuronal cellular membrane surface, could lead to an increase in MTsat. However, *in vitro* studies have shown contradictory results. Studies on hiPSC-derived astrocytes from ALD patients showed that they were unable to support the co-cultured neurons, resulting in reduced morphological complexity and impaired dendritic arbourization.^43^ Accordingly, impaired neuritogenesis in AALD patients should result in a decrease in MT signal in the GM.

A further hypothesis is that MTsat correlates not only with the neuronal but also with the glial membrane surface in the GM. Reactive astrogliosis leads to hypertrophy of astrocyte processes, potentially increasing the surface area of the astrocyte membrane.^55,56^ As outlined in more detail above, several studies have indicated glial cell activation in ALD. Astrocytes differ between the GM and WM and adapt to the requirements of their local environment. Protoplasmic astrocytes reside in the cortical GM, have a bushy appearance and carry numerous branched processes. Fibrous astrocytes, in contrast, are predominantly found in the WM and possess fewer processes.^57,58^ Therefore, astrocyte activation in the GM might cause a greater increase in membrane surface area compared to the WM, resulting in a more significant rise in MTsat in the GM than in the WM. This would explain why we do not observe a significant increase in MTsat in the frontal WM, although the increased Ins indicates astrocyte activation.

Also, the process of microglia activation itself could lead to an increase in MTsat. A study that analysed regional correlations of MTsat in the cortex with transcriptome data found a correlation between the MTsat signal and the expression of microglia-specific genes in the cortex.^51^ They proposed that this relationship was due to ‘off-resonance saturation’ in the neighbourhood of iron-rich microglia, although the MTsat signal is not typically considered iron sensitive.

In summary, activation of both astrocytes and microglia could potentially lead to an increase in the MTsat signal in the GM and WM of AALD patients.

### Spatial distribution of MRI parameter changes may reflect different aspects of disease involvement

The effect sizes for WMV and cortical MTsat differed in regional analyses; the frontal WM and the frontal GM were most affected. In addition, MRS showed an increase in Ins in the frontal WM. One possible explanation is that the spatial gradients of gene expression, like the variable expression of ALD protein in different oligodendrocyte populations in the WM with high levels, e.g. in the corpus callosum,^41^ play a role in the spatial distribution of the effects. Microvascular perfusion disturbances in ABCD1 deficiency without cerebral manifestations also demonstrate regional differences and are most prevalent in the splenium of the corpus callosum, possibly rendering these WM regions more vulnerable.^59^ Interestingly, the primary localization of CALD lesions in the WM upon manifestation is age dependent: in childhood, demyelination in CALD typically starts to manifest in the PO WM or concurrently in the frontal WM. In adolescence, CALD lesions predominantly appear in the frontal or cerebellar WM, whereas in adulthood, they primarily affect the corticospinal tracts.^60^ This leads to the hypothesis that the regional differences in our MRI findings correlate with an enhanced vulnerability of the frontal WM during adolescence in AALD. Future studies should investigate whether elevated WMV, altered WM diffusivity, elevated WM Ins or increased cortical MTsat can predict CALD development, and whether similar changes are present in the occipital lobe in younger patients.

## Conclusion

In summary, our quantitative MRI findings, obtained for the first time in clinically asymptomatic adolescents with ALD, reveal early pathological processes in both WM and GM that are already active prior to the onset of symptoms. Whereas some of our MRS and DTI findings in AALD confirm previous observations of WM pathology in AMN or in the NAWM of CALD patients, others are novel. Specifically, the increase in WMV and the increased MTsat values in the cortex represent novel MRI phenotypes for this condition. Considering the MR parameters as a whole, it appears plausible that dysmyelination, astrocytosis and microgliosis are already present in the brains of adolescent AALD patients. Whether these early changes act as predisposing factors for the development of the inflammatory and neurodegenerative processes leading to CALD or AMN remains to be determined.

As is common in studies of rare diseases, our study is subject to methodological limitations. Prospective studies in AALD patients and age-matched healthy controls are needed to confirm these quantitative MRI findings. Including a larger age range would enable characterization of the temporal evolution of microstructural alterations in AALD. Furthermore, conducting retrospective volumetric studies of structural MRI data from patients who later developed CALD could help determine whether early WMV hypertrophy is associated with an increased risk of developing CALD.

For adult-onset neurodegenerative diseases, such as Parkinson’s or Alzheimer’s disease, it is well established that patients undergo prodromal phases that begin decades before the onset of clinical symptoms. Effective neuroprotective therapies, therefore, target these pre-symptomatic stages.^61,62^ Our findings suggest that ALD may similarly involve a prodromal phase, during which the brain is already vulnerable to neurodegeneration or neuroinflammation. With the expansion of newborn screening programmes for ALD, the number of diagnosed pre-symptomatic ALD patients is expected to increase, creating new opportunities for early intervention. A better understanding of the molecular mechanisms underlying early microstructural brain alterations in ALD may facilitate the development of neuroprotective therapies aimed at reducing the risk of CALD or delaying the onset of AMN. The multimodal MRI biomarkers identified in this study may, therefore, play an important role in future early-phase clinical trials and intervention strategies.

### Limitations

ALD, a rare disease, occurs with an estimated minimum frequency of 1:21 000 hemizygous males in the USA.^63^ Before the implementation of newborn screening programmes, neurologically asymptomatic patients were typically identified either through family screening when an index patient tested positive or when adrenal insufficiency became apparent. The patients in our cohort were recruited under these circumstances. Additionally, we had to deal with artefacts (especially movement artefacts) in our MRI data. We implemented a conservative strategy to exclude noisy data, at the cost of sample size. Moreover, not all imaging modalities were acquired consistently across MRI sessions, with only a subset of modalities available for the majority of sessions. Therefore, one limitation of our dataset is the small sample size. Additionally, the number of control subjects was relatively small. For site-specific parametrization of braincharts.io centiles, only 34 recordings from non-matched control subjects were used, whereas braincharts.io suggests 100 control subjects. Given the small number of subjects per group and the uneven number of longitudinal recordings at mismatched sampling time points, a linear mixed-effects model was employed. This approach is particularly suitable for analysing longitudinal neuroimaging datasets with similar cohort characteristics.^64^ It has been applied previously to analyse longitudinal datasets in rare diseases, such as metachromatic leukodystrophy and neuronal ceroid lipofuscinosis.^65–67^ The linear mixed-effects models showed a good fit for the fixed variables in most volumetric, DTI and MTsat measurements, while the model fit for the MRS data was comparatively weak. In addition, the MRS data were acquired across two scanner upgrade versions. Although previous studies suggest that MRS measurements are not affected by MRI upgrades,^26^ and our own analysis likewise showed no significant effects on metabolites, the MRS findings should nevertheless be interpreted with caution. The interpretation of our results may be further limited by potential selection bias associated with single-centre recruitment, by scanner-related variability resulting from MRI upgrades throughout the study and by the exclusion of recordings or data points determined to be of inadequate quality or designated as outliers.

## Supplementary Material

fcag311_Supplementary_Data

## Data Availability

The data that support the findings of this study are available to qualified investigators for non-commercial academic purposes by contacting the corresponding author. The data are not publicly available to protect the privacy of research participants. The code for processing the MTsat maps can be found at https://github.com/helms-man/MTsat-processing-FSL. The R-scripts that were used to analyse the data and the scripts used to analyse the DTI data are in the Supplementary material.
