# Supplementary material for: Multimodal quantitative MRI finds early brain changes in asymptomatic X-linked adrenoleukodystrophy
Source: Brain Commun. 2026 Aug 29;8(5):fcag311. doi: 10.1093/braincomms/fcag311 (PMC13550773; doi:10.1093/braincomms/fcag311)
Supplement: Supplementary file 1 [file fcag311_supplementary_data.zip › Supplementary_Materials.pdf]

Supplementary methods

MRI acquisition

Structural imaging

Structural imaging utilized a vendor-provided T1-weighted (T1w) 3D MPRAGE sequence with an isotropic resolution of 1 mm (repetition time (TR)/ inversion time (TI)/ echo time (TE) = 2250/900/3.26 msec).

Diffusion tensor imaging

For diffusion tensor imaging (DTI), a TurboSTEAM sequence was employed (46 slices, 2.2 mm isotropic resolution, 24 directions at b = 950 s/mm2 and 1 reference at b = 0 s/mm2).[1] Up to three measurements were taken to enhance the signal-to-noise ratio (SNR) through averaging.

Magnetization transfer imaging

Whole-brain MT imaging was performed using a 3D FLASH sequence with 1.25 mm isotropic resolution and 240 mm field-of-view employing partial acquisition techniques. MT contrast was imposed on a proton density reference (TR/TE/α = 25/4.9 msec/5°) by applying a 12.8 msec Gaussian MT-pulse of 540 nominal flip angle, 2.2 kHz off resonance before excitation. Using a second T1w reference (TR/α = 11 msec/15°), maps of the percentage MT saturation (MTsat) were calculated as previously described.[2]

Quantitative single-voxel 1H MR spectroscopy

1H MR spectra (64 accumulations) were acquired in standard regions in the parieto-occipital WM (WM PO) or frontal lobe WM (WM FL). VOIs of 4.1 mL were selected using a STEAM localization sequence with TR/TE/mixing time = 6000/20/10 ms as described previously with the use of an 8-channel or, more recently, a 64-channel head coil.[3]

Data analysis

MRI volumetry

For volumetry, we used only images obtained by the Magnetom Tim TRIO system, as scanner updates are known to influence volumetric measures.[4] Brain volume segmentation utilized the recon-all segmentation pipeline in the FreeSurfer toolbox version 6

(http://surfer.nmr.mgh.harvard.edu/).[5] For quality control, the T1-weighted images were rated for image quality into three classes (good, moderate, and bad) based on visual rating criteria.[6] Scans with bad image quality were excluded from further analysis. We further excluded scans that had more than 100 surface holes in the Freesurfer parcellation, as the number of topological defects correlates with image quality.[7] To analyze normalized brain volumes, we divided them by the estimated total intracranial volume (eTIV).

To calculate lobe-specific cortical GM and subcortical WM volumes, we grouped the Freesurfer parcellation volumes according to their affiliation with cortical lobes, as outlined in the Freesurfer documentation (https://surfer.nmr.mgh.harvard.edu/fswiki/CorticalParcellation), and summed the bilateral volumes. Cingulate structures were categorized into either the parietal or frontal lobes. Unsegmented WM and the corpus callosum were classified as deep WM.

Volumetric measurements of our dataset were compared with an open source volumetry dataset that was similarly processed with Freesurfer 6, for input to braincharts.[8] We calculated ventricle size in this dataset as the difference of BrainSegVol and BrainSegVolNotVent, as in our dataset. Recordings of male subjects aged 10 – 18 years with good quality, as indicated by the quality control datasheet of the external dataset, were included in the comparison.

Preprocessing of DTI data

The QSIprep boilerplate information describes the processing steps: For anatomical data preprocessing, the T1-weighted (T1w) image was corrected for intensity non-uniformity (INU) using N4BiasFieldCorrection, and used as an anatomical reference throughout the workflow. The anatomical reference image was reoriented into AC-PC alignment via a 6-DOF transform extracted from a full Affine registration to the MNI152NLin2009cAsym template. A full nonlinear registration to the template from AC-PC space was estimated via symmetric nonlinear registration (SyN) using antsRegistration. Brain extraction was performed on the T1w image using SynthStrip and automated segmentation was performed using SynthSeg from FreeSurfer version 7.3.1. Up to 3 DWI series were concatenated, with preprocessing operations performed on the concatenated DWI series. MP-PCA denoising, as implemented in MRtrix3's dwidenoise, was applied with a 5-voxel window. After MP-PCA, the mean intensity of the DWI series was adjusted so all the mean intensity of the b=0 images matched across each separate DWI scanning sequence. FSL's (version 6.0.5.1) eddy was used for head motion correction and Eddy current correction. Eddy was configured with a q-space smoothing factor of 10, a total of 5 iterations, and 1000 voxels used to estimate hyperparameters. A linear first level model and a linear second

level model were used to characterize Eddy current-related spatial distortion. q-space coordinates were forcefully assigned to shells. Field offset was attempted to be separated from subject movement. Shells were aligned post-eddy. Eddy's outlier replacement was run. Data were grouped by slice, only including values from slices determined to contain at least 250 intracerebral voxels. Groups deviating by more than 4 standard deviations from the prediction had their data replaced with imputed values. Final interpolation was performed using the jac method. Several confounding time-series were calculated based on the preprocessed DWI: framewise displacement (FD) using the implementation in Nipype. The head-motion estimates calculated in the correction step were also placed within the corresponding confounds file. Slicewise cross correlation was also calculated. The DWI time-series were resampled to ACPC, generating a preprocessed DWI run in ACPC space with 2.2mm isotropic voxels.

Quality control and analysis of DTI data

We excluded both recordings that had either missing WM areas, as identified by visual inspection, or bad slices, as determined by QSIprep. In addition, QSIprep metrics, such as contrast-to-noise ratio (CNR), neighboring DWI correlation (NDC), and framewise displacement (FD), were employed for further quality evaluation. Recordings with CNR and NDC values that fell 1.5 interquartile ranges (IQR) below the first quartile or recordings with FD that exceeded 1.5 IQR above the third quartile were also excluded.

The tract-based spatial statistics (TBSS) workflow contained in FSL (version 6.0.7) was adapted to calculate average DTI measures for WM tracts as defined by the JHU ICBM-DTI-81 atlas.[9–11] All subjects' FA data were aligned to the ICBM-DTI-81 atlas FA image by nonlinear registration. The ICBM-DTI-81 atlas labels were registered to the FA images of each subject by inverse registration matrices from the previous step. Median FA, MD, AD, and RD values for total supratentorial WM, commissural, projection and association tracts were obtained with fslmaths and averaged for bilateral tracts. We used the ICBM-DTI-81 atlas to summarize the tracts; for commissural tracts we used labels 3 (Genu CC), 4 (Body CC), 5 (Splenium CC), 49 + 50 (Tapetum); for projection tracts we used labels 15 + 16 (Corticospinal), 17 + 18 (Medial lemniscus), 19 + 20 (Inferior cerebellar peduncle), 21 + 22 (Superior cerebellar peduncle), 23 + 24 (Cerebral peduncle), 25 + 26 (Anterior limb of internal capsule), 27 + 28 (Posterior limb of internal capsule), 29 + 30 (Retrolenticular part of internal capsule); for association tracts we used labels 6 (Fornix), 31 + 32 (Sagittal stratum), 33 + 34 (External capsule), 35 + 36 (Cingulum), 37 + 38 (Cingulum hippocampus), 39 + 40 (Fornix cres/Stria terminalis), 41 + 42 (Superior longitudinal fasciculus), 43 + 44 (Superior fronto-occipital fasciculus), 45 + 46 (Inferior fronto-

occipital fasciculus), 47 + 48 (Uncinate fasciculus). For total supratentorial WM we used all commissural, projection and association tracts.

Quality control and analysis of MTsat data

MTsat maps were registered to brain-extracted T1-weighted images from the Freesurfer volumetry workflow (described in the MRI volumetric section) by affine registration with 6 degrees of freedom using FLIRT (FSL version 6.0.7.6). Regions from aseg segmentations of T1-weighted images were then mapped onto the MTsat map to extract region-wise median MTsat values using fslstats.

The quality of MTsat images and their overlay with the Freesurfer aseg segmentation was visually assessed and rated (good, moderate, and bad). MTsat images that scored “bad” (either showed artifacts or did not overlap well with the Freesurfer segmentation) were excluded from further analysis.

Statistical analysis

Group differences between AALD and control subjects were analyzed using linear mixed-effects models. The models were fitted using the restricted maximum likelihood (REML) approach. Before the analysis, the dependent variables (e.g., volumetry results or DTI parameters) and eTIV were scaled in standard deviations (SD) of the mean, and age was demeaned. For volumetric analysis, we used group, age, and eTIV as fixed effects and subject as a random effect [formula: dependent_variable ~ group + age + eTIV + (1 | subject)]. For MTsat, MRS and DTI data analysis, group and age were considered as fixed effects, and subject was considered as a random effect [formula: dependent_variable ~ group + age + (1 | subject)]. P-values and confidence intervals were calculated using Satterthwaite approximations, as this produces acceptable type 1 error rates, also for smaller sample sizes.[12] False discovery rate (FDR) adjusted p-values (q-values) were calculated by the Benjamini-Hochberg procedure for group effects for multiregional measurements. For volumetry and atlas based MTsat analysis we applied FDR separately on global volumes (i.e. WM, GM, Ventricles) and regional volumes (lobar WM or GM, subcortical GM; major tract classes for DTI). The linear mixed model performance was analyzed by checking for singularity and calculating marginal and conditional Nakagawa R2 values as well as unadjusted and adjusted intraclass correlation coefficients (ICC).

WM volume centiles were determined by calculating first the median volume centile from the longitudinal recordings of each subject. Second, a one-sample Wilcoxon signed-rank test was performed to evaluate whether the median centiles of AALD or control differ from 0.5. We did

not perform a multiple testing correction when comparing AALD and control data against the median value of 0.5, as the hypothesis of interest tested whether the patient group deviates from the 0.5 baseline. The analysis of the control group against the same baseline was performed as a methodological validation, rather than a secondary related hypothesis. Accordingly, these two tests address distinct inferential questions and do not constitute a family of dependent comparisons. Therefore, we did not expect Type I error rate inflation that would necessitate correction across these analyses.

For comparison of our volumetry data with the external dataset we first compared the volumetry data of the healthy controls with a linear model to find batch effects, including age as a covariate; for this analysis the volumetry measurements were not scaled [formula: dependent_variable ~ age + Dataset]. Then we used a linear mixed model to compare the volumetry data of our cohort with the external cohorts [formula: dependent_variable ~ group + age + Dataset + (1| subject)].

References

1. Nolte UG, Finsterbusch J, Frahm J. Rapid isotropic diffusion mapping without susceptibility artifacts: whole brain studies using diffusion-weighted single-shot STEAM MR imaging. *Magn Reson Med*. 2000;44(5):731-736. doi:10.1002/1522-2594(200011)44:5<731::aid-mrm11>3.0.co;2-1

2. Helms G, Dathe H, Kallenberg K, Dechent P. High-resolution maps of magnetization transfer with inherent correction for RF inhomogeneity and T1 relaxation obtained from 3D FLASH MRI. *Magn Reson Med*. 2008;60(6):1396-1407. doi:10.1002/mrm.21732

3. Frahm J, Michaelis T, Merboldt K, et al. Localized NMR spectroscopy In vivo. Progress and problems. *NMR Biomed*. 1989;2(5-6):188-195. doi:10.1002/nbm.1940020504

4. Plitman E, Bussy A, Valiquette V, et al. The impact of the Siemens Tim Trio to Prisma upgrade and the addition of volumetric navigators on cortical thickness, structure volume, and 1H-MRS indices: An MRI reliability study with implications for longitudinal study designs. *Neuroimage*. 2021;238. doi:10.1016/j.neuroimage.2021.118172

5. Fischl B. FreeSurfer. *Neuroimage*. 2012;62(2):774-781. doi:10.1016/j.neuroimage.2012.01.021

6. Backhausen LL, Herting MM, Buse J, Roessner V, Smolka MN, Vetter NC. Quality control of structural MRI images applied using FreeSurfer-a hands-on workflow to rate motion artifacts. *Front Neurosci*. 2016;10(DEC). doi:10.3389/fnins.2016.00558

7. Rosen AFG, Roalf DR, Ruparel K, et al. Quantitative assessment of structural image quality. *Neuroimage*. 2018;169:407-418. doi:10.1016/j.neuroimage.2017.12.059

8. Zughayyar I, Bauer M, Güttler C, et al. A FastSurfer Database for Age-Specific Brain Volumes in Healthy Children: A Tool for Quantifying Localized and Global Brain Volume Alterations in Pediatric Patients. *Brain Behav*. 2025;15(7). doi:10.1002/brb3.70689
Data downloaded on OSF.io under https://osf.io/kj7hy/ in February 2026.

9. Smith SM, Jenkinson M, Johansen-Berg H, et al. Tract-based spatial statistics: Voxelwise analysis of multi-subject diffusion data. *Neuroimage*. 2006;31(4):1487-1505. doi:10.1016/j.neuroimage.2006.02.024

10. Smith SM, Jenkinson M, Woolrich MW, et al. Advances in functional and structural MR image analysis and implementation as FSL. In: *NeuroImage*. Vol 23. 2004. doi:10.1016/j.neuroimage.2004.07.051

11. Mori S, Oishi K, Jiang H, et al. Stereotaxic white matter atlas based on diffusion tensor imaging in an ICBM template. *Neuroimage*. 2008;40(2):570-582. doi:10.1016/j.neuroimage.2007.12.035

12. Luke SG. Evaluating significance in linear mixed-effects models in R. *Behav Res Methods*. 2017;49(4):1494-1502. doi:10.3758/s13428-016-0809-y

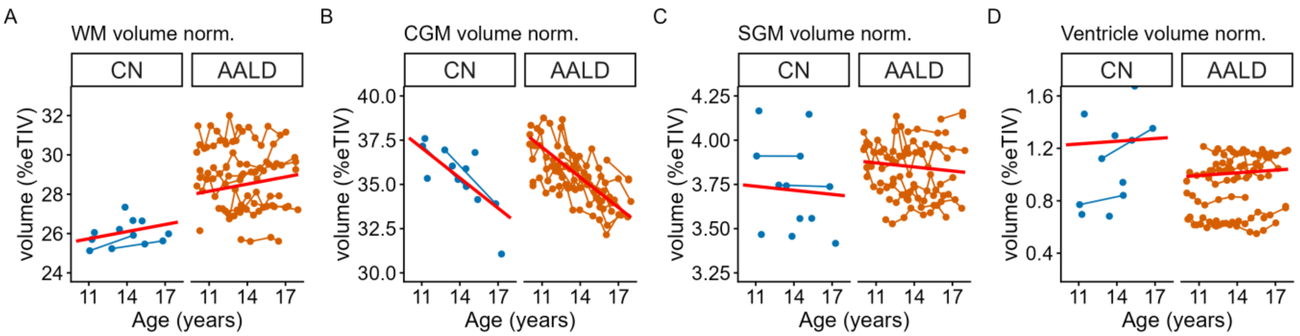

Supplementary Fig.1 Longitudinal normalized brain structure volumes. Longitudinal normalized volume measurements of white matter (WM, A), cortical gray matter (CGM, B), subcortical gray matter (SGM, C) volume and ventricle volume (D) from controls (CN, n = 10 subjects, 12 recordings, 1-2 recordings per subject) and asymptomatic adrenoleukodystrophy (AALD) patients (n = 12 subjects, 88 recordings, 1-14 recordings per subject). Volumes were normalized (norm.) in percent of the estimated total intracranial volume (eTIV). Each dot represents a measurement. Connected dots indicate repeated measurements from the same individual. The red line illustrates the group specific intercept and the age slope of the linear mixed-effects model analysis. The results of the linear mixed effects model analysis are shown in Supplementary Table 2.

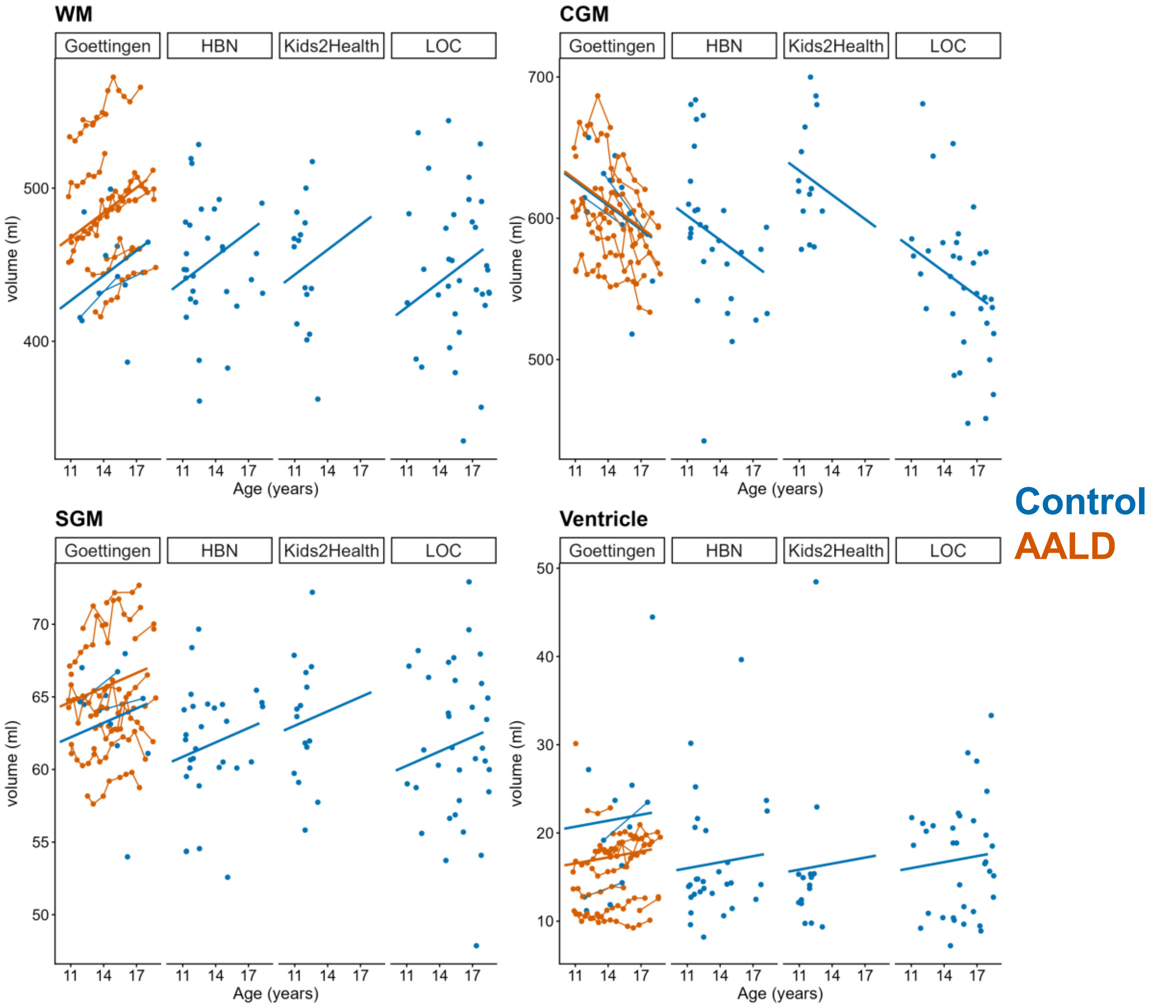

Supplementary Fig.2 Longitudinal brain structure volumes in comparison with external datasets. Longitudinal volume measurements of white matter (WM), cortical gray matter (CGM), subcortical gray matter (SGM) volume and ventricle volume from male controls (CN) and asymptomatic adrenoleukodystrophy (AALD) patients between age 10 and 18 years. Each dot represents a measurement. Connected dots indicate repeated measurements from the same individual. The lines illustrate the group specific intercept and the age slope of the linear mixed model analysis. The data from our controls (Dataset "Goettingen", CN, n = 10 subjects, 12 recordings, 1-2 recordings per subject) and AALD patients (Dataset "Goettingen", n = 12 subjects, 88 recordings, 1-14 recordings per subject) and from the external datasets [Datasets "HBN" (n = 29), "Kids2Health" (n = 15), "LOC" (n = 34)] are displayed on separated panels. The results of the linear mixed effects model analysis are shown in Supplementary Table 3 (only comparison between control datasets with a linear model) and 4 (including AALD patients with a linear mixed model).

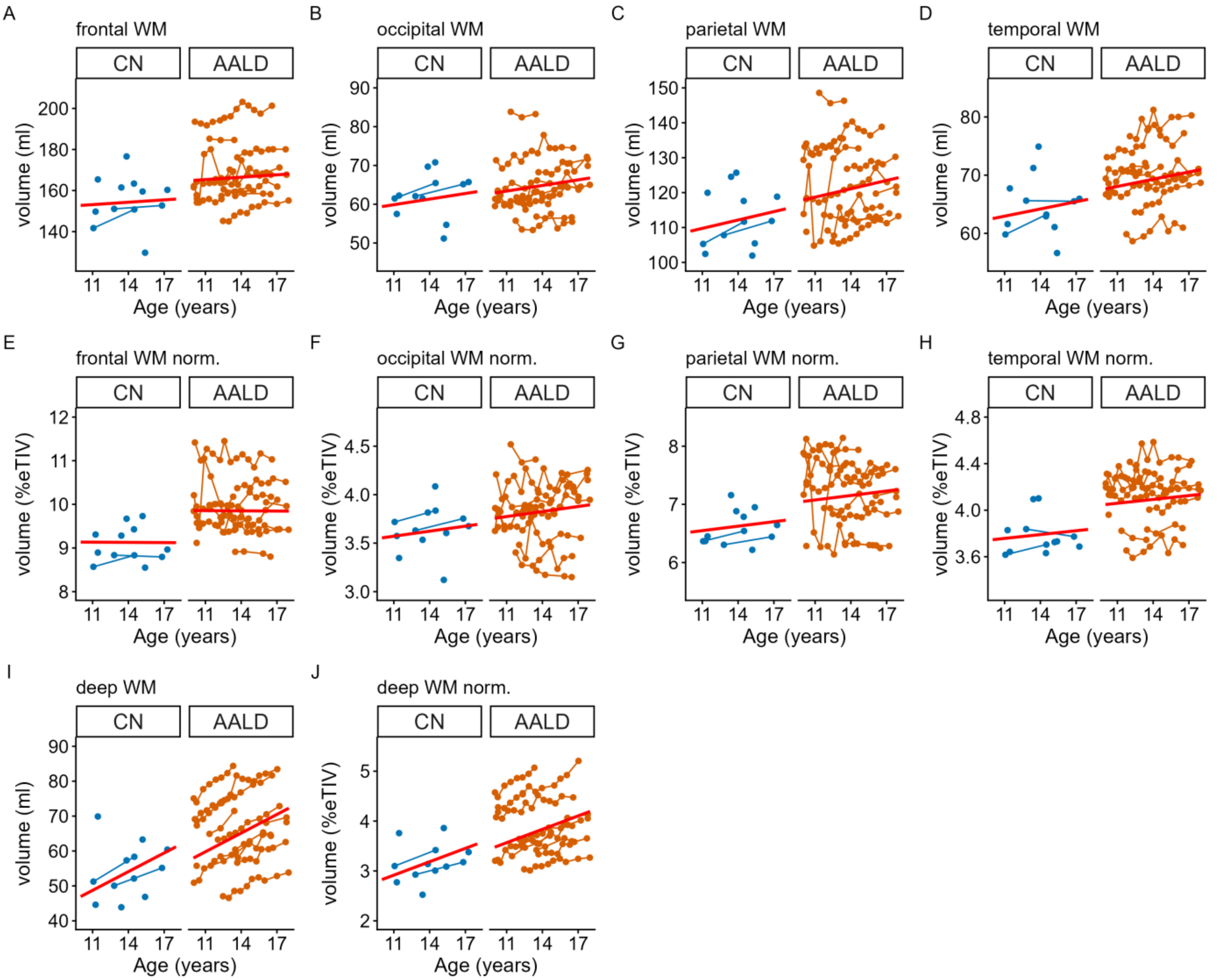

Supplementary Fig.3 Longitudinal regional white matter volumes. Longitudinal measurements of regional white matter (WM) volume from controls (CN, n = 10 subjects, 12 recordings, 1-2 recordings per subject) and asymptomatic adrenoleukodystrophy (AALD) patients (n = 12 subjects, 88 recordings, 1-14 recordings per subject). **(A-D, I)** Raw volumes. **(E – H, J)** Volumes were normalized (norm.) in percent of the estimated total intracranial volume (eTIV). Each dot represents a measurement. Connected dots indicate repeated measurements from the same individual. The red line illustrates the group specific intercept and the age slope of the linear mixed model analysis. The results of the linear mixed effects model analysis are shown in Supplementary Table 2.

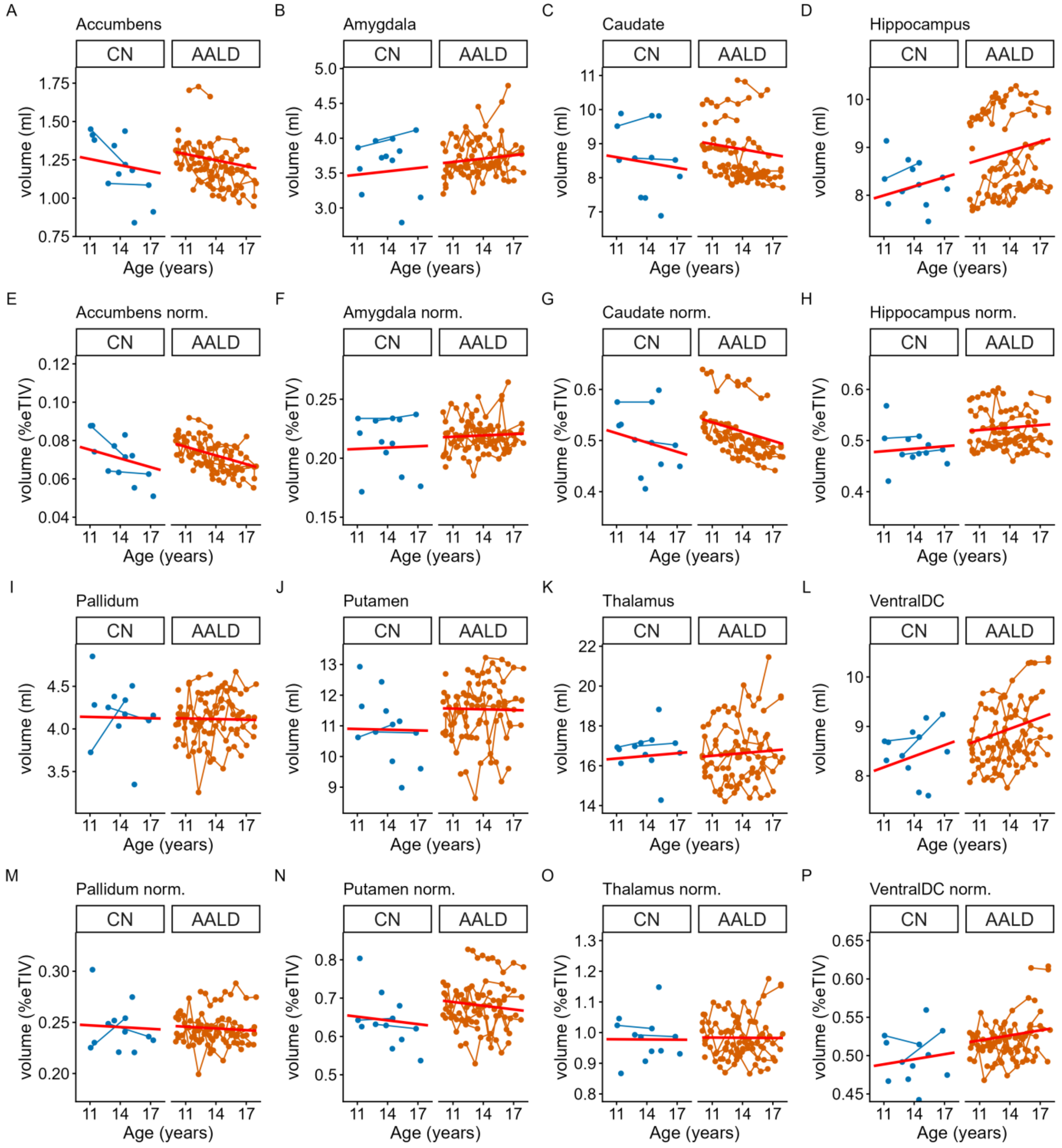

Supplementary Fig.4 Longitudinal regional subcortical gray matter volumes. Longitudinal measurements of regional subcortical gray matter region volumes from controls (CN, n = 10 subjects, 12 recordings, 1-2 recordings per subject) and asymptomatic adrenoleukodystrophy (AALD) patients (n = 12 subjects, 88 recordings, 1-14 recordings per subject). **(A-D, I-L)** Raw volumes. **(E-H, M-P)** Volumes were normalized (norm.) in percent of the estimated total intracranial volume (eTIV). Each dot represents a measurement. Connected dots indicate repeated measurements from the same individual. The red line illustrates the group specific intercept and the age slope of the linear mixed model analysis. The results of the linear mixed effects model analysis are shown in Supplementary Table 2. VentralDC = ventral diencephalon.

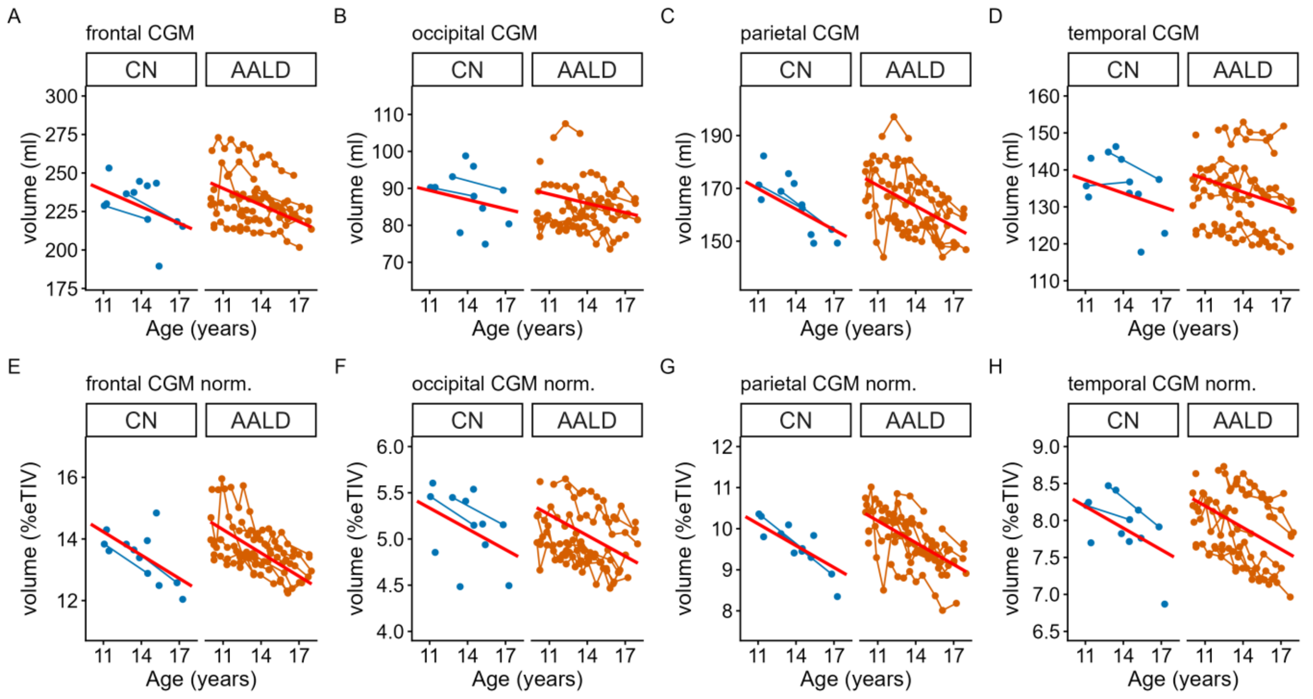

Supplementary Fig.5 Longitudinal brain region volumes. Longitudinal measurements volumes from controls (CN, n = 10 subjects, 12 recordings, 1-2 recordings per subject) and asymptomatic adrenoleukodystrophy (AALD) patients (n = 12 subjects, 88 recordings, 1-14 recordings per subject) in regional cortical gray matter (CGM). **(A-D)** Raw volumes. **(E-H)** Volumes were normalized (norm.) in percent of the estimated total intracranial volume (eTIV). Each dot represents a measurement. Connected dots indicate repeated measurements from the same individual. The red line illustrates the group specific intercept and the age slope of the linear mixed model analysis. The results of the linear mixed effects model analysis are shown in Supplementary Table 2.

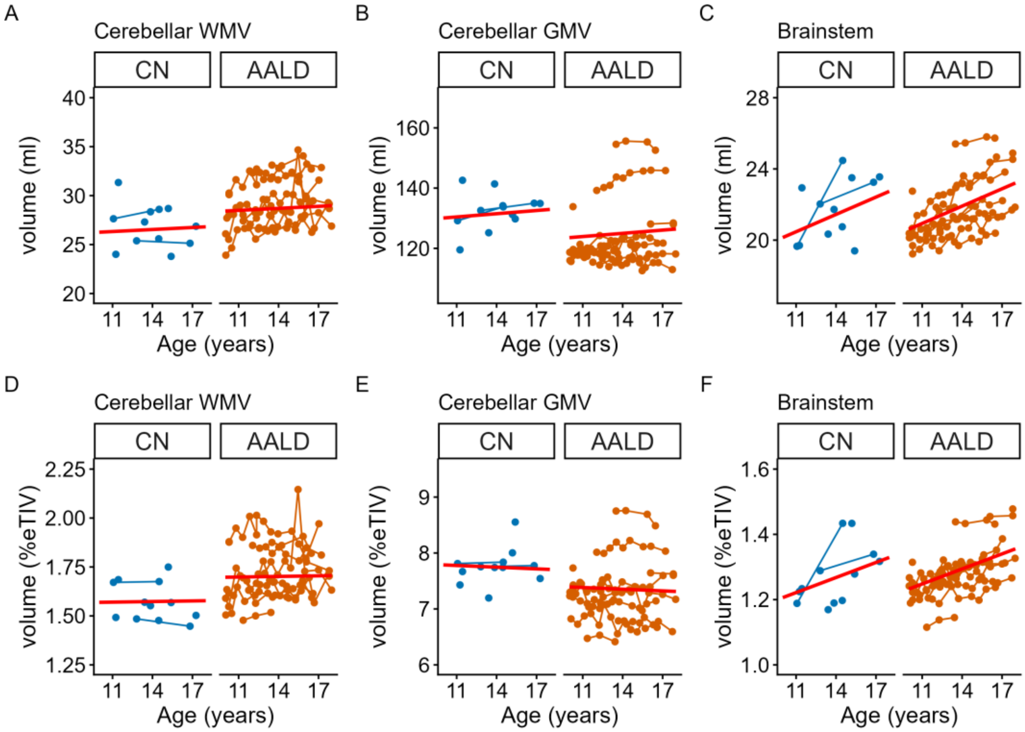

Supplementary Fig.6 Longitudinal brain region volumes. Longitudinal measurements volumes from controls (CN, n = 10 subjects, 12 recordings, 1-2 recordings per subject) and asymptomatic adrenoleukodystrophy (AALD) patients (n = 12 subjects, 88 recordings, 1-14 recordings per subject) for cerebellar white matter volume (WMV), cerebellar gray matter volume (GMV) and brainstem. **(A-C)** Raw volumes. **(D-F)** Volumes were normalized (norm.) in percent of the estimated total intracranial volume (eTIV). Each dot represents a measurement. Connected dots indicate repeated measurements from the same individual. The red line illustrates the group specific intercept and the age slope of the linear mixed model analysis. The results of the linear mixed effects model analysis are shown in Supplementary Table 2.

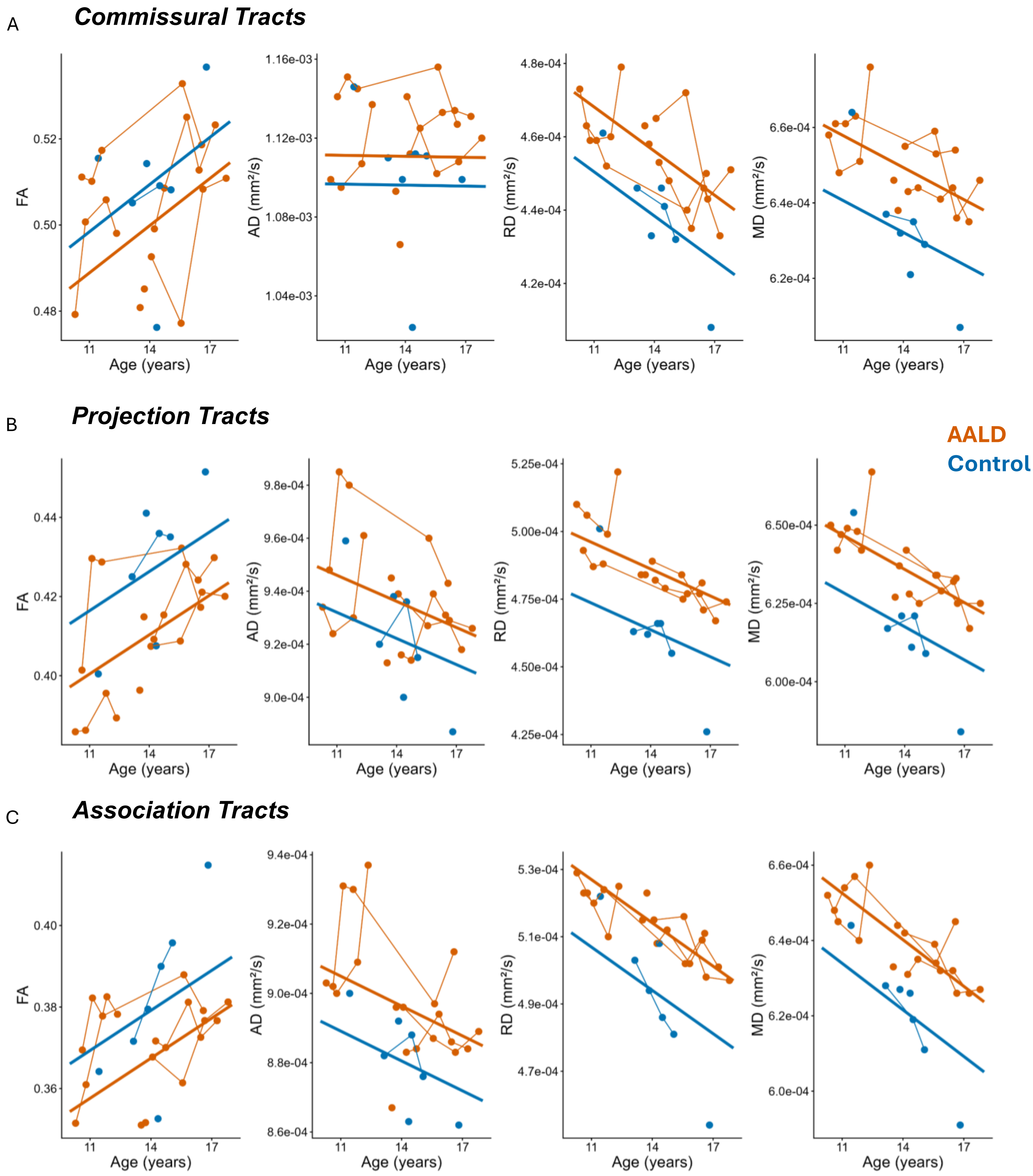

Supplementary Fig.7 Longitudinal diffusion tensor imaging (DTI) measurements. Longitudinal measurements of fractional anisotropy (FA), axial diffusivity (AD), radial diffusivity (RD) and mean diffusivity (MD) of control subjects (n = 5, 7 recordings, 1 subject with 3 recordings) and asymptomatic adrenoleukodystrophy (AALD) patients (n = 6, 20 recordings, 1 – 5 recordings per patient) in commissural tracts (A), projection tracts (B) and association tracts (C). Each dot represents a measurement. Connected dots with thin lines indicate repeated measurements from the same individual. The thick lines illustrate the group specific intercept and the age slope of the linear mixed model analysis. The results of the linear mixed effects model analysis of DTI in major white matter tract classes are shown in Supplementary Table 6.

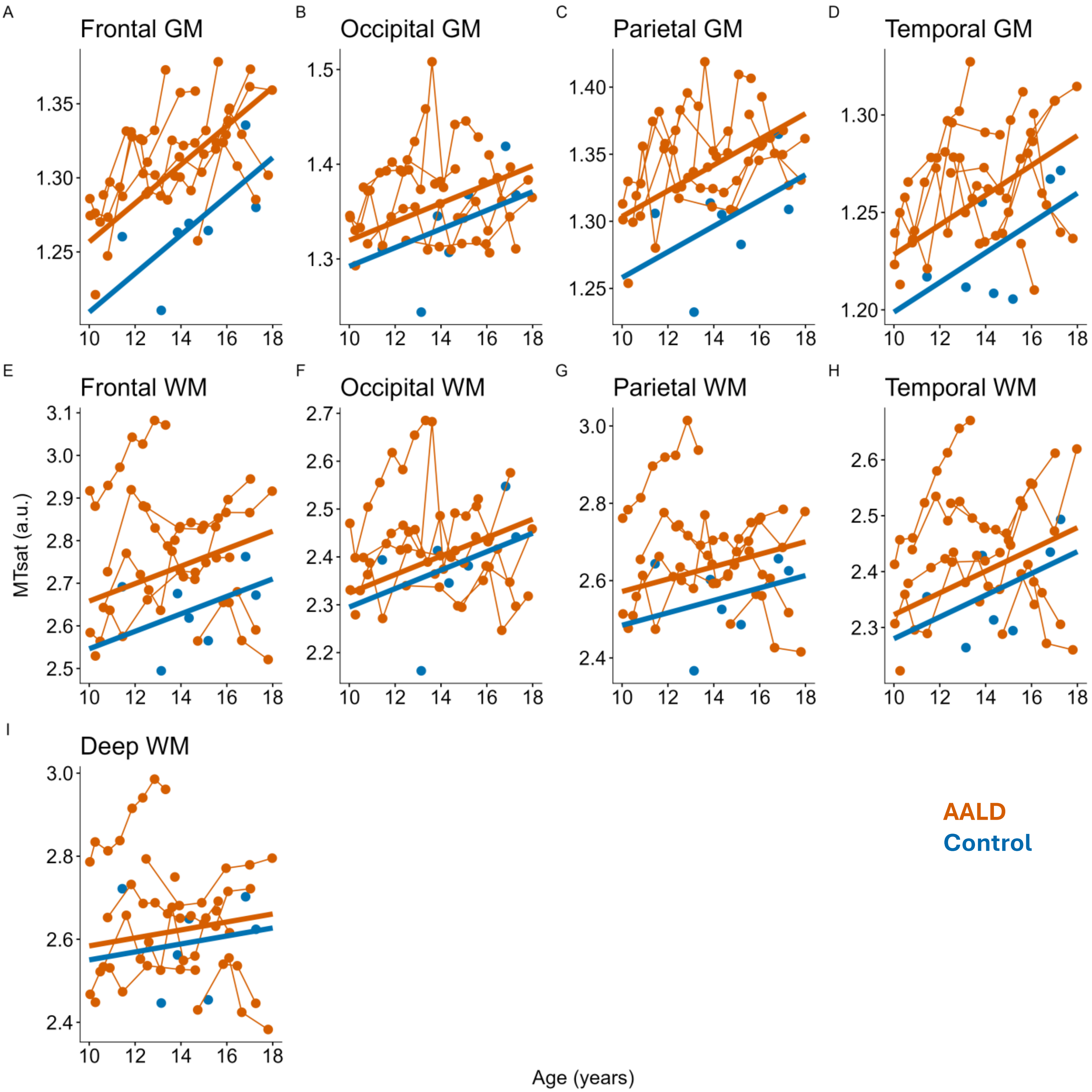

Supplementary Fig.8 Longitudinal magnetization transfer saturation (MTsat) measurements. Longitudinal measurements of MTsat in asymptomatic adrenoleukodystrophy (AALD) patients (n = 10, 53 recordings, 1-11 recordings per subject) and controls (n=7, 7 recordings) in regional gray matter (GM, **A-D)** and white matter (WM, **E-I)**. MTsat was measured in arbitrary units (a.u.). Each dot represents a measurement. Connected dots with thin lines indicate repeated measurements from the same individual. The thick lines illustrate the group specific intercept and the age slope of the linear mixed model analysis. The results of the MTsat linear mixed effects model analysis are shown in Supplementary Table 7.

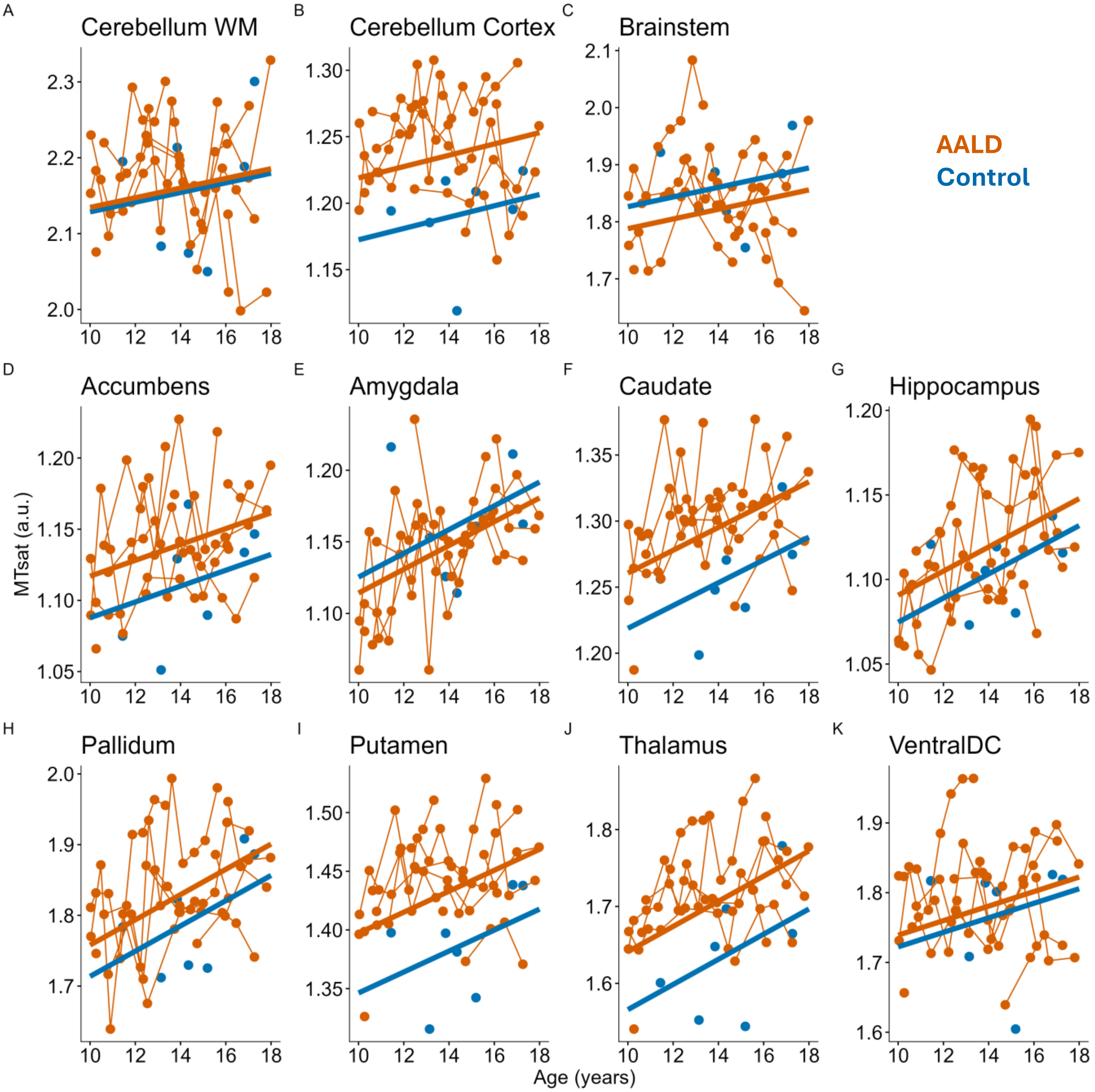

Supplementary Fig.9 Longitudinal magnetization transfer saturation (MTsat) measurements. Longitudinal measurements of MTsat from asymptomatic adrenoleukodystrophy (AALD) patients (n = 10, 53 recordings, 1-11 recordings per subject) and controls (n=7, 7 recordings) in cerebellum white matter **(A)** and cortex **(B)**, brainstem **(C)** and subcortical structures **(D-K)**. Each dot represents a measurement. Connected dots with thin lines indicate repeated measurements from the same individual. The thick lines illustrate the group specific intercept and the age slope of the linear mixed model analysis. The results of the MTsat linear mixed effects model analysis are shown in Supplementary Table 7. VentralDC = ventral diencephalon.

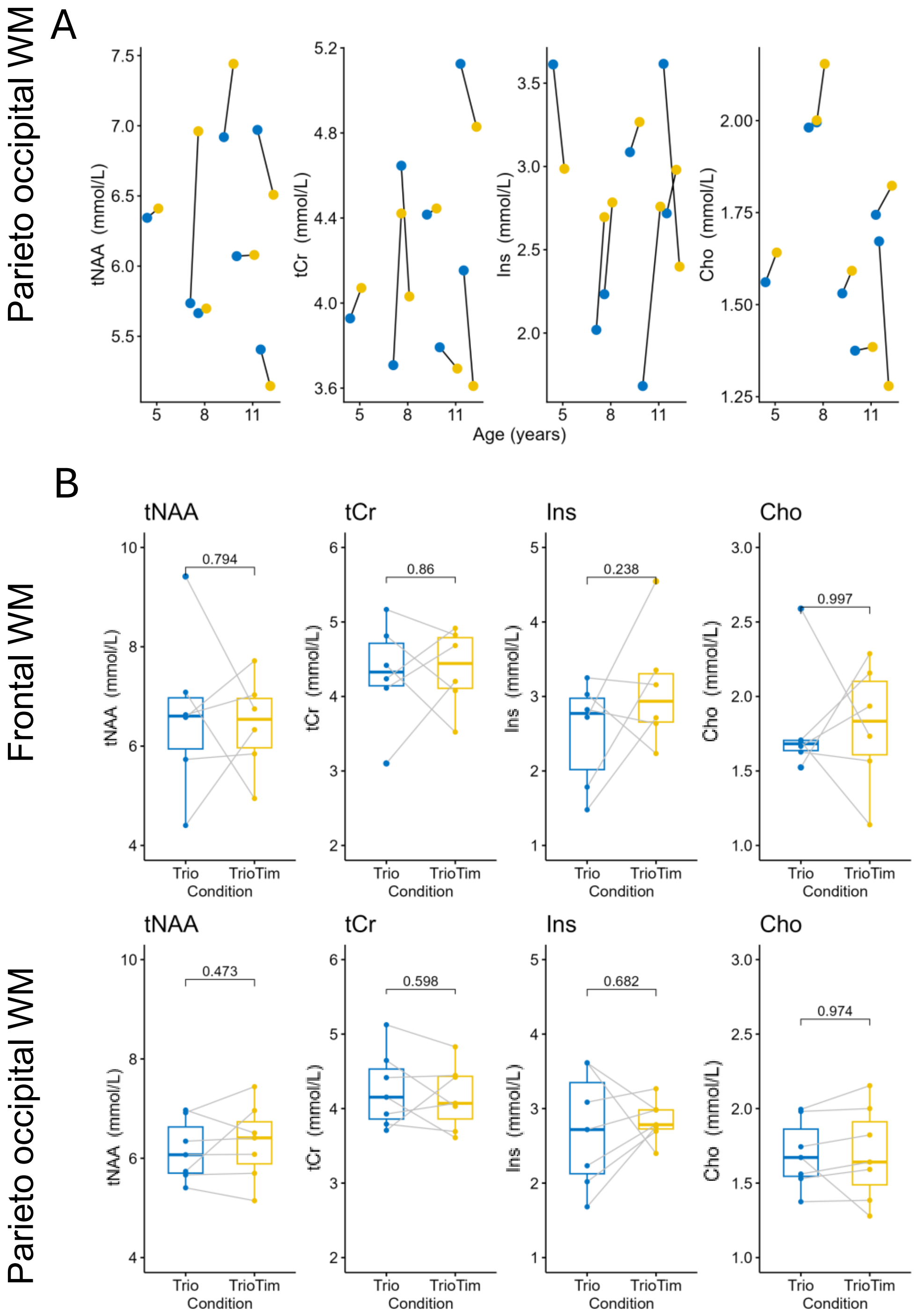

Supplementary Fig.10 Impact of scanner upgrade on MR spectroscopy (MRS). (A) MRS measurements before (blue) and after (yellow) the upgrade of the MR scanner from Trio to TrioTim, showing total N-acetyl aspartate (tNAA), total creatine (tCr), choline (Cho) and inositol (Ins) in parieto-occipital white matter (WM). For this analysis younger asymptomatic adrenoleukodystrophy (AALD) patients (n=7) were also included. We show only parieto-occipital WM values to illustrate the age distribution of recordings. Each dot represents one measurement. Connected dots with thin lines indicate repeated measurements from the same individual. Age is indicated in years, metabolite concentrations in mmol/L. **(B)** Boxplots of the measurements in AALD patients before and after the MR scanner upgrade in the frontal (n=6) and parieto-occipital (n=7) WM. Each datapoint represents a measurement before (blue) or after (yellow) the upgrade from Trio to TrioTim, connected datapoints are from the same subject. P-values from paired t-tests are indicated on the plots with squared brackets. There were no significant differences in the metabolite measurements. The full results of the paired t-test analyses are shown in Supplementary Table 8.

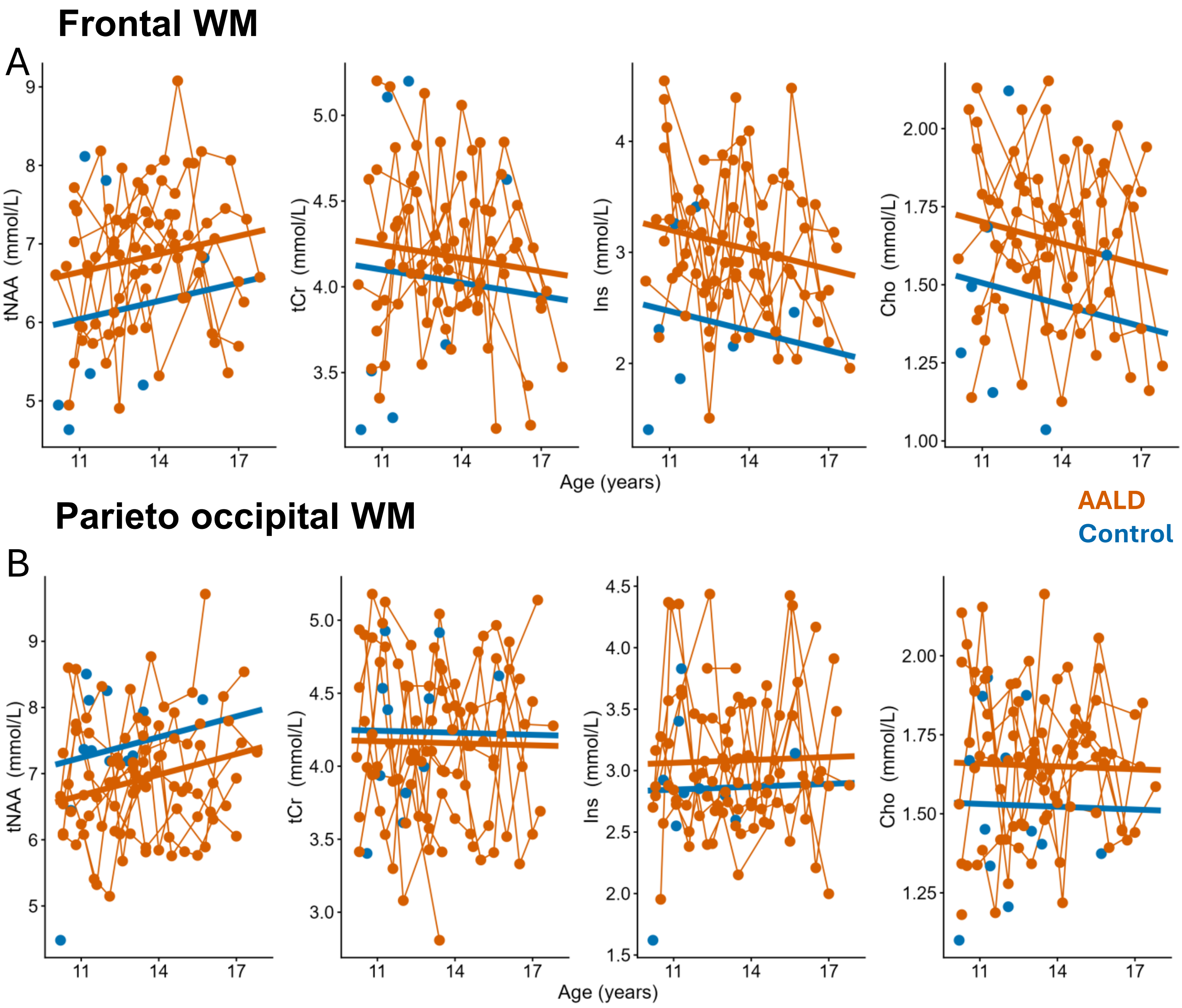

Supplementary Fig.11 Longitudinal MR spectroscopy (MRS) data. Longitudinal measurements of total N-acetyl aspartate (tNAA), total creatine (tCr), inositol (Ins) and choline (Cho) in frontal (**A**, 7 controls, 10 AALD patients) and parieto-occipital (**B**, 10 controls, 11 AALD patients) white matter (WM) of asymptomatic adrenoleukodystrophy (AALD) patients and control subjects. Each dot represents a measurement. Connected dots with thin lines indicate repeated measurements from the same individual. The thick lines illustrate the group specific intercept and the age slope of the linear mixed-effects model analysis. The results of the linear mixed effects model analysis are shown in Supplementary Table 9.
